# Host-pathogen interaction between pitaya and *Neoscytalidium dimidiatum* reveals the mechanisms of immune response associated with defense regulators and metabolic pathways

**DOI:** 10.1186/s12870-023-04685-y

**Published:** 2024-01-02

**Authors:** Meng Wang, Zhouwen Wang, Yi Ding, Shaoling Kang, Senrong Jiang, Zhuangjia Yang, Zhan Xie, Jialin Wang, Shuangshuang Wei, Jiaquan Huang, Dongdong Li, Xingyu Jiang, Hua Tang

**Affiliations:** 1 School of Breeding and Multiplication, Hainan University, Sanya, 572025 China; 2 School of Tropical Agriculture and Forestry, Hainan University, Haikou, 570228 China; 3 Yazhou Bay Laboratory, Sanya, 572025 China; 4 College of Life Sciences, Hainan University, Haikou, 570228 China; 5 National Center of Technology Innovation for Saline-Alkali Tolerant Rice/College of Coastal Agricultural Sciences, Guangdong Ocean University, Zhanjiang, 524088 China

**Keywords:** Host–pathogen interaction, Pitaya canker, *N.dimidiatum*, Transcriptomics

## Abstract

**Background:**

Understanding how plants and pathogens regulate each other's gene expression during their interactions is key to revealing the mechanisms of disease resistance and controlling the development of pathogens. Despite extensive studies on the molecular and genetic basis of plant immunity against pathogens, the influence of pitaya immunity on *N. dimidiatum* metabolism to restrict pathogen growth is poorly understood, and how *N. dimidiatum* breaks through pitaya defenses. In this study, we used the RNA-seq method to assess the expression profiles of pitaya and *N. dimidiatum* at 4 time periods after interactions to capture the early effects of *N. dimidiatum* on pitaya processes.

**Results:**

The study defined the establishment of an effective method for analyzing transcriptome interactions between pitaya and *N. dimidiatum* and to obtain global expression profiles. We identified gene expression clusters in both the host pitaya and the pathogen *N. dimidiatum*. The analysis showed that numerous differentially expressed genes (DEGs) involved in the recognition and defense of pitaya against *N. dimidiatum*, as well as *N. dimidiatum*’s evasion of recognition and inhibition of pitaya. The major functional groups identified by GO and KEGG enrichment were responsible for plant and pathogen recognition, phytohormone signaling (such as salicylic acid, abscisic acid). Furthermore, the gene expression of 13 candidate genes involved in phytopathogen recognition, phytohormone receptors, and the plant resistance gene (*PG*), as well as 7 effector genes of *N. dimidiatum*, including glycoside hydrolases, pectinase, and putative genes, were validated by qPCR. By focusing on gene expression changes during interactions between pitaya and *N. dimidiatum*, we were able to observe the infection of *N. dimidiatum* and its effects on the expression of various defense components and host immune receptors.

**Conclusion:**

Our data show that various regulators of the immune response are modified during interactions between pitaya and *N. dimidiatum*. Furthermore, the activation and repression of these genes are temporally coordinated. These findings provide a framework for better understanding the pathogenicity of *N. dimidiatum* and its role as an opportunistic pathogen. This offers the potential for a more effective defense against *N. dimidiatum*.

**Supplementary Information:**

The online version contains supplementary material available at 10.1186/s12870-023-04685-y.

## Background

Pitaya (*Hylocereus polyrhizus*), a tropical and subtropical fruit native to Latin America, is popular among consumers for its high content of vitamin C and minerals such as calcium and phosphorus [1]. China has recently surpassed Vietnam as the world's largest pitaya producer in terms of land area. However, pitaya production is often affected by biotic and abiotic stresses, leading to serious consequences on the yield and quality of pitaya [2, 3]. Among these stresses, pitaya canker caused by *N. dimidiatum* has become the primary factor limiting the development of the pitaya industry [4, 5].

*N. dimidiatum* belongs to the class *Dothideomycetes* and the *Botryosphaeriaceae* family, known for being destructive blight and canker pathogens of plants [6]. *N. dimidiatum* colonizes the young stems of pitaya, causing them to turn from green to yellow and develop brown spots. These spots continue to spread throughout the plantation and can ultimately destroy the entire pitaya, resulting in an annual loss of approximately 27 million USD in China [5, 7–9]. Currently, controlling pitaya canker is very difficult, and only the use of broad-spectrum fungicides can effectively restrict its spread. With increasing awareness of environmental protection, there is an increasing emphasis on screening and breeding disease-resistant varieties of pitaya, as well as controlling pitaya canker in a rational and scientific manner. Understanding the pathogenesis of plant diseases and the effective functioning of the plant's immune system is currently one of the key areas of research in plant science.

Molecular genetic studies have revealed a set of patterns of action of plant immunity and downstream immune signaling components within plant cells [10, 11]. Plants commonly employ a multi-layered monitoring system to defend against pathogenic fungi. This system includes mechanisms such as plasma membrane localization and intracellular immune receptor recognition of non-self or modified self [12]. Surface pattern recognition receptors (PRRs) act as the initial sensors to detect pathogen-associated molecular patterns (PAMPs) and trigger a basal resistance response known as pattern-triggered immunity (PTI) [11]. To evade plant defenses and cause plant pathogenesis, fungal pathogens have developed a range of virulence molecules that inhibit and disrupt PTI responses at various stages of the host's immune system [11]. Similarly, plants have evolved intracellular nucleotide-binding domains and leucine-rich repeat (NLR) receptors to monitor effectors and activate effector triggered immunity (ETI) in plants [10]. They also share several downstream signaling molecules, such as ethylene (ET), salicylic acid (SA), jasmonic acid (JA), and abscisic acid (ABA). These molecules signaling pathways that regulate transcriptional reprogramming in plants [13]. Additionally, pathogenic fungi encounter rapid changes in the external environment and host resistance responses during plant infection. These responses include stomatal closure [14], activation of mitogen-activated protein kinases (MAPKs) [15, 16], bursts of reactive oxygen species (ROS), deposition of callose and lignin, induction of phytohormones, and expression of transcription factors (TFs) and pathogenesis-related proteins (PRs) [10, 13]. As a result, changes in the interaction and gene expression patterns of both the pathogen and the plant are triggered, leading to adaptations for survival. Despite extensive research on plant-pathogen interactions, little is known to date about how *N. dimidiatum* triggers pitaya canker and how effectively the pitaya immune system monitors and limits *N. dimidiatum* infection.

To investigate the interaction between *N. dimidiatum* and pitaya, with a focus on identifying key events that may contribute to pitaya infection. We utilized a transcriptome-based approach to characterize the interaction between *N. dimidiatum* and pitaya, aiming to reveal key events that may lead to pitaya infection. We used the RNA-seq to quantify the level of coordinated expression, which provides an unbiased method for analyzing the transcriptomes of both species. By combining the published genome of pitaya [17] with *N. dimidiatum* genomic data [9], we were able to accurately analyze the transcriptomes of both pitaya and *N. dimidiatum*. This allows us to assess how *N. dimidiatum* breaks through pitaya's defenses to promote disease development and when pitaya recognizes *N. dimidiatum* through specific signaling pathways to resist pathogenic infestation. Here, we define the overall gene expression profiles of *N. dimidiatum* and the host pitaya, identify differential gene clusters, and reveal the virulence-related genes in *N. dimidiatum*, as well as the host’s defense mechanisms.

## Results

### Establishment of a transcriptome approach for pitaya and *N. dimidiatum*

We examined the progression of pitaya infection by *N. dimidiatum* over time using microscopy analysis (Fig. S1). Our results showed that *N. dimidiatum* could induce mild disease symptoms in pitaya at 5 days post-inoculation (dpi). Based on these findings, we have chosen 5 dpi as the initial sampling time point for studying the interaction between pitaya and *N. dimidiatum*. We selected 10 cm-old pitaya stems for cultivation and observed pitaya shoots with a length of approximately 20 cm after 30 days. The spore suspension of *N. dimidiatum* was evenly sprayed on the surface of pitaya shoots. Pitaya samples infected at a depth of 0.2–0.3 cm below the surface (including the epidermis) were collected. This ensured that the fungi samples accounted for the proportion of all mRNAs. These samples were then used to prepare libraries for RNA-seq analysis. This method successfully enriched the sequence of *N. dimidiatum* and enabled us to obtain high-quality transcriptome data from pitaya samples of *N. dimidiatum*.

### RNA-seq data profile of pitaya and *N. dimidiatum*

**Fig. 1:**
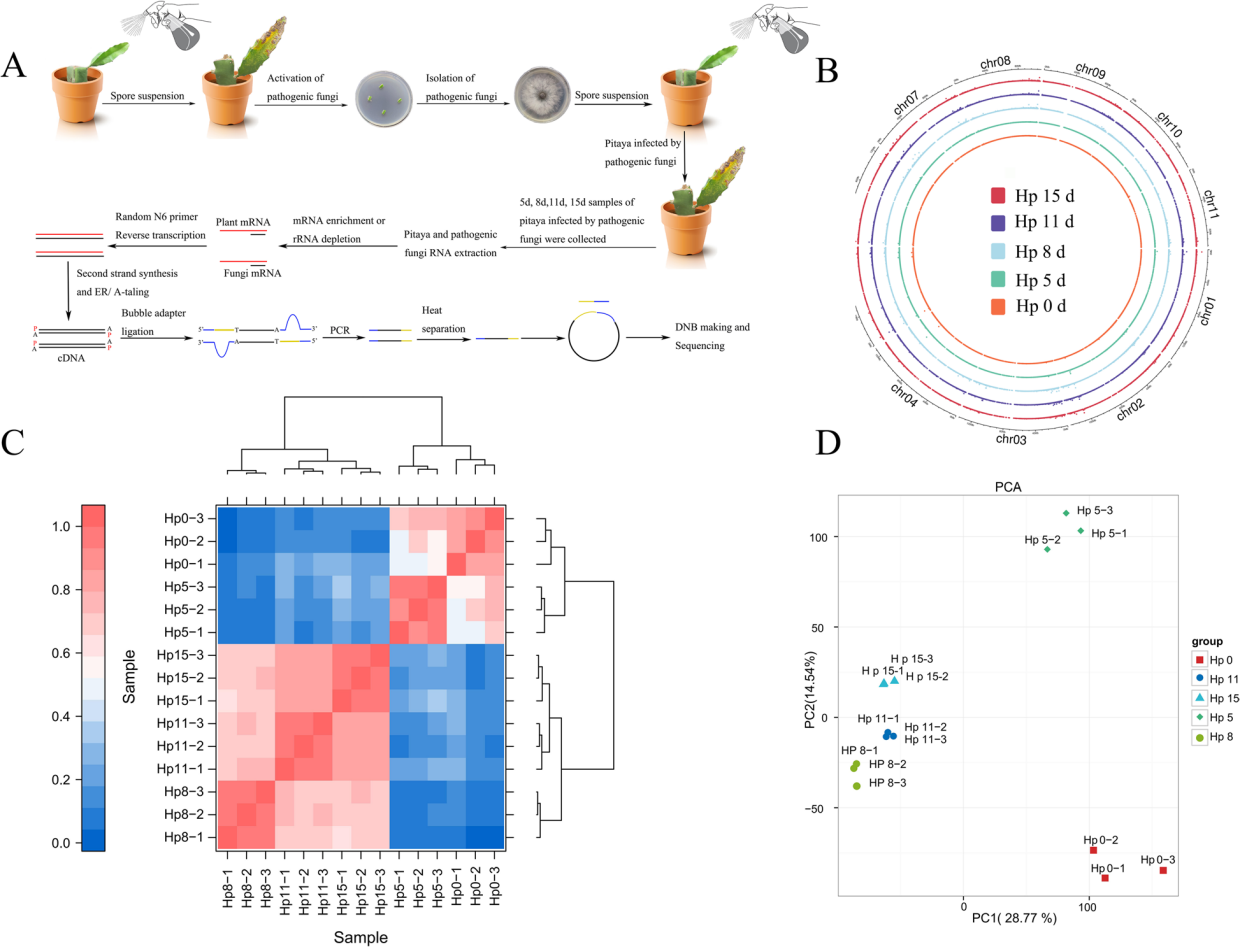
Flow chart of the transcriptome and overall data report for the interaction between pitaya and *N. dimidiatum*. **A** Spore suspensions were sprayed on pitaya shoots, and infected pitaya samples were collected at 5 days, 8 days, 11 days, and 15 days after spraying for cDNA library preparation and sequencing, respectively. **B** The circos plot shows gene expression values per kilobase of the transcript. **C** The heat map displays the correlation of expression between samples. The horizontal and vertical coordinates of the plot are sample numbers, the order of which is determined by the sample correlation clustering results, and the top and right sides of the plot are the corresponding clustering trees; the colors reflect the magnitude of correlation between samples. **D** The image shows the principal component analysis (PCA) of the pitaya

To investigate the gene expression characteristics during the dynamic infection of *N. dimidiatum* in pitaya, we conducted RNA-seq analysis to examine the dynamics of the interaction between pitaya and *N. dimidiatum*. Healthy pitaya and *N. dimidiatum* spores were collected as controls. Infected pitaya stems were collected and photographed at 5 days, 8 days, 11 days, and 15 days, respectively (Fig. S2). Three biological replicates of each sample were used to extract total RNA, which was sequenced on the Illumina HiSeq 2000 platform (Fig. 1A). The total pitaya samples were sequenced using Illumina-based deep sequencing yielding ~ 234 million cDNA reads. ~ 89 million reads could be mapped to the pitaya genome with a coverage of over 84%. ~ 128 million reads could be localized to the genome of *N. dimidiatum*, covering 0.5%-38.63%. This provides adequate coverage for reliable RNA-seq analysis (Table S1, Fig. 1B). According to the principal component analysis (PCA) of the interactions between pitaya and *N. dimidiatum*, the biological replicates of each group were correlated and clustered together (Fig. 1C, D, Fig. S3), indicating that the RNA-seq data was highly stable.

### Overall assessment of the expression profiles in pitaya and *N. dimidiatum* genes

**Fig. 2:**
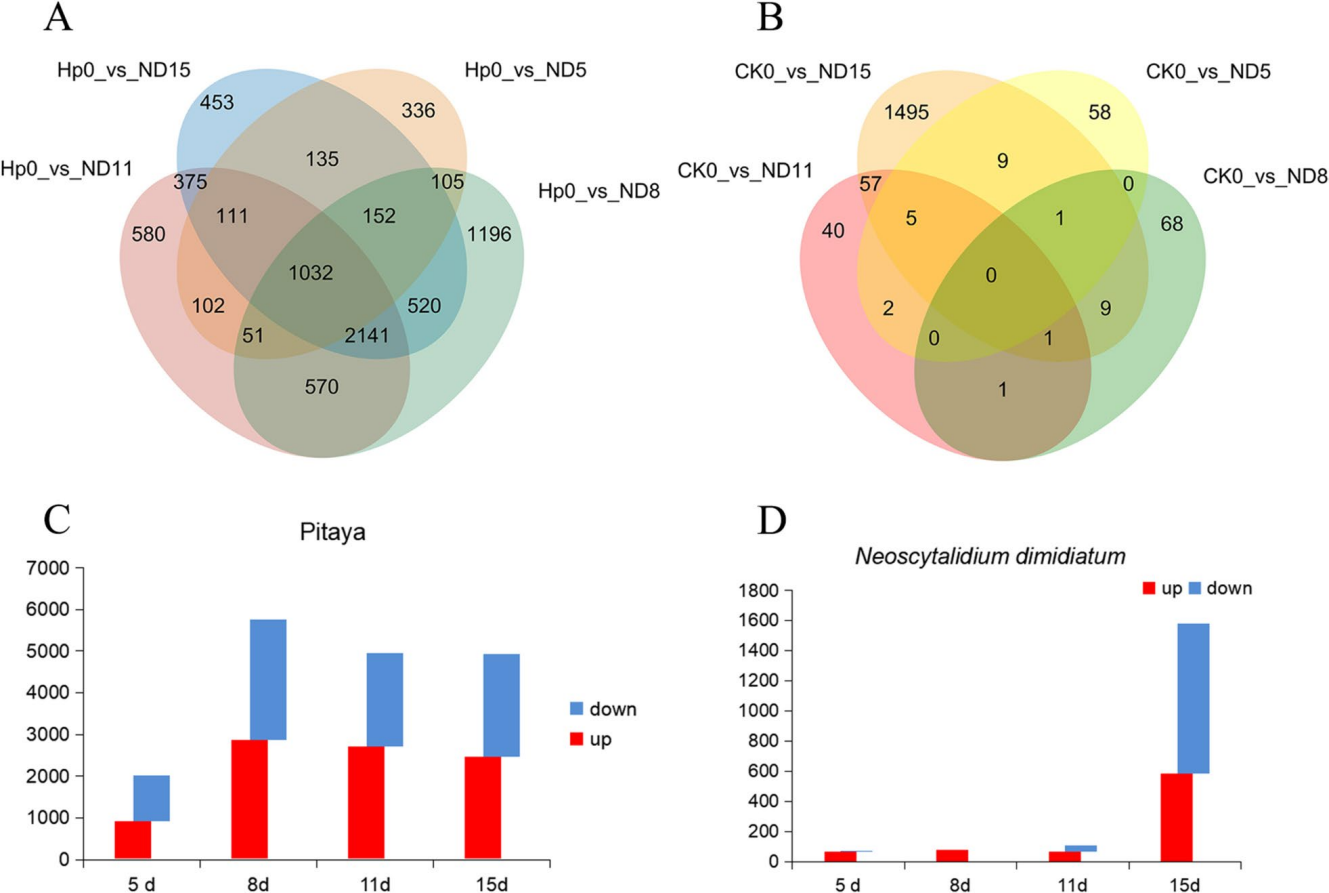
The number of DEGs identified at various stages of the interaction between pitaya and *N. dimidiatum*. **A** The number of DEGs in pitaya identified at different stages of the interaction with *N. dimidiatum*. The overlapping circles indicate the DEGs shared among different groups. Hp0: the number of DEGs for non-infected pitaya. ND5, ND8, ND11, and ND15 represent the 5, 8, 11, and 15 days of the pitaya-*N. dimidiatum* interaction, respectively. **B** The number of DEGs in *N. dimidiatum* identified at different stages of the interaction with pitaya. CK0: the number of DEGs for *N. dimidiatum* spores. **C** The number of upregulated and downregulated genes in pitaya during the interaction stage between pitaya and *N. dimidiatum*. The blue color indicates the number of down-regulated genes; the red color indicates the number of up-regulated genes. **D** The number of upregulated and downregulated genes in *N. dimidiatum* during the interaction stage between pitaya and *N. dimidiatum*

In this study, we used the DESeq2_edgeR software to calculate differentially expressed genes (DEGs), FC = 2, and a false discovery rate (FDR) of 0.01. First, the DEGs of pitaya were counted. There were 2,024 DEGs at 5 days, with 929 up-regulated and 1,095 were down-regulated. There were 5,765 DEGs, with 2,859 up-regulated and 2,908 down-regulated at 8 days. There were 4,962 DEGs, with 2,702 up-regulated and 2,260 down-regulated at 11 days. Similarly, at 15 days, there were 4,919 DEGs, with 2,454 up-regulated and 2,465 down-regulated (Fig. 2A, C). To investigate the DEGs of *N. dimidiatum* after colonizing on pitaya, we compared the *N. dimidiatum* samples with its library. At 5 days, there were 75 DEGs, with 69 up-regulated and 6 down-regulated. At 8 days, there were 80 DEGs, all of which were upregulated. At 11 days, there were 106 DEGs, with 67 up-regulated and 39 were 1,577 DEGs, with 588 up-regulated and 989 down-regulated (Fig. 2B, D).

### *N.dimidiatum* induced host responses

#### Expression modulation at 5 days

**Fig. 3:**
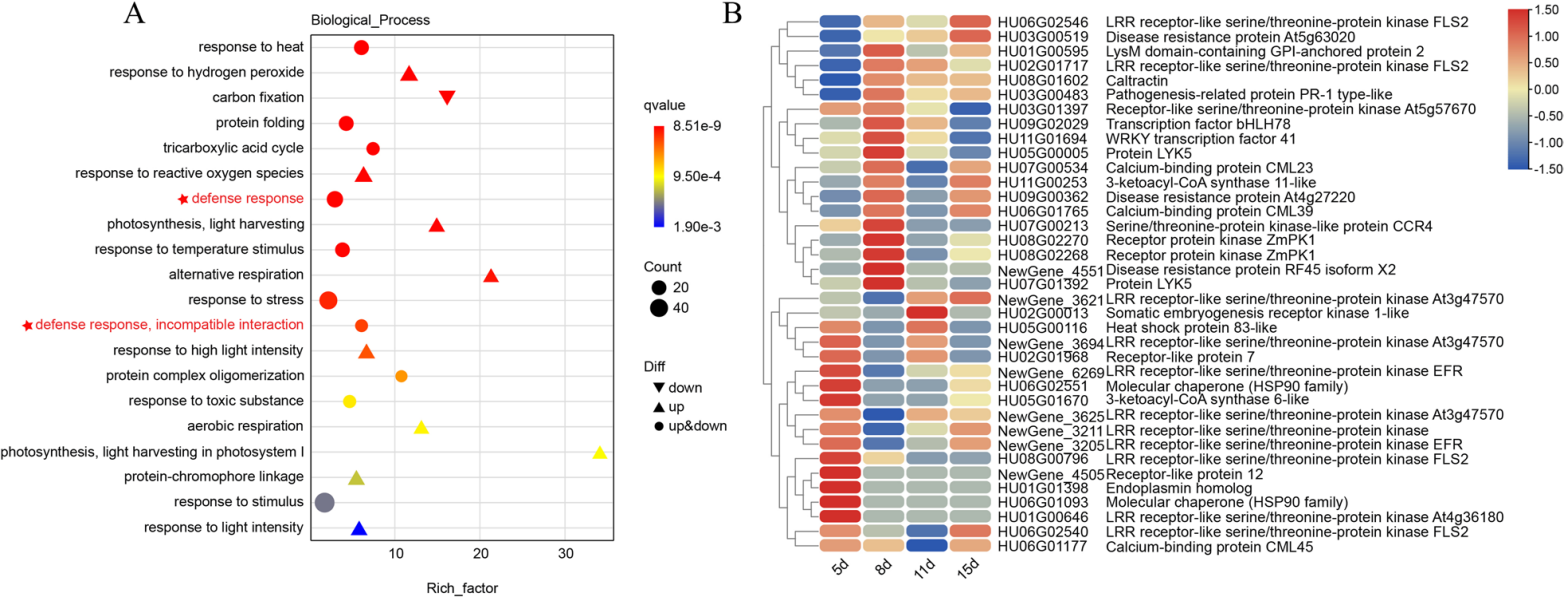
Pitaya infected with *N. dimidiatum* Go enrichment and heatmap analysis at 5 days. **A** GO enrichment of pitaya infested with *N. dimidiatum* at 5 d. **B** Pitaya infected with *N. dimidiatum* by phytopathogenic fungi interactions heatmap analysis at 5 d

We analyzed RNA-seq data of pitaya 5 days after *N. dimidiatum* infection (log2FC > 2), and the results revealed that pitaya may be able to recognize and respond to *N. dimidiatum* at 5 days (Fig. 3A). To further confirm this, we performed a heatmap analysis and annotation of these enriched genes. The analysis revealed that many PRRs on the cell surface, which are involved in interactions with plant pathogens, showed increased expression levels (Fig. 3B). Analysis of the KEGG pathway showed that these receptors interact with RAR1 and SGT1 to indirectly elicit hypersensitivity reactions (HR) in pitaya. Notably, we found that the gene for Hsp90 in the folded protein was enriched and upregulated (Table S2). Hsp90 is a molecular chaperone of SGT1 that is required for the maturation of NLRs73 in various plants. It amplifies signals through cascade reactions and phosphorylation, leading to ETI responses in plants [13]. Additionally, we noticed that several of the enriched genes showed significantly upregulated expression at 8 days (Fig. 3B), such as *LYK5*, *LysM*, *PR-1*, *WRKY41*, and the receptor protein ZmPK1. The reason may be that the shift of pitaya's own defense strategy and signaling pathway occurred at 8 days.

### Expression modulation at 8 days

**Fig. 4:**
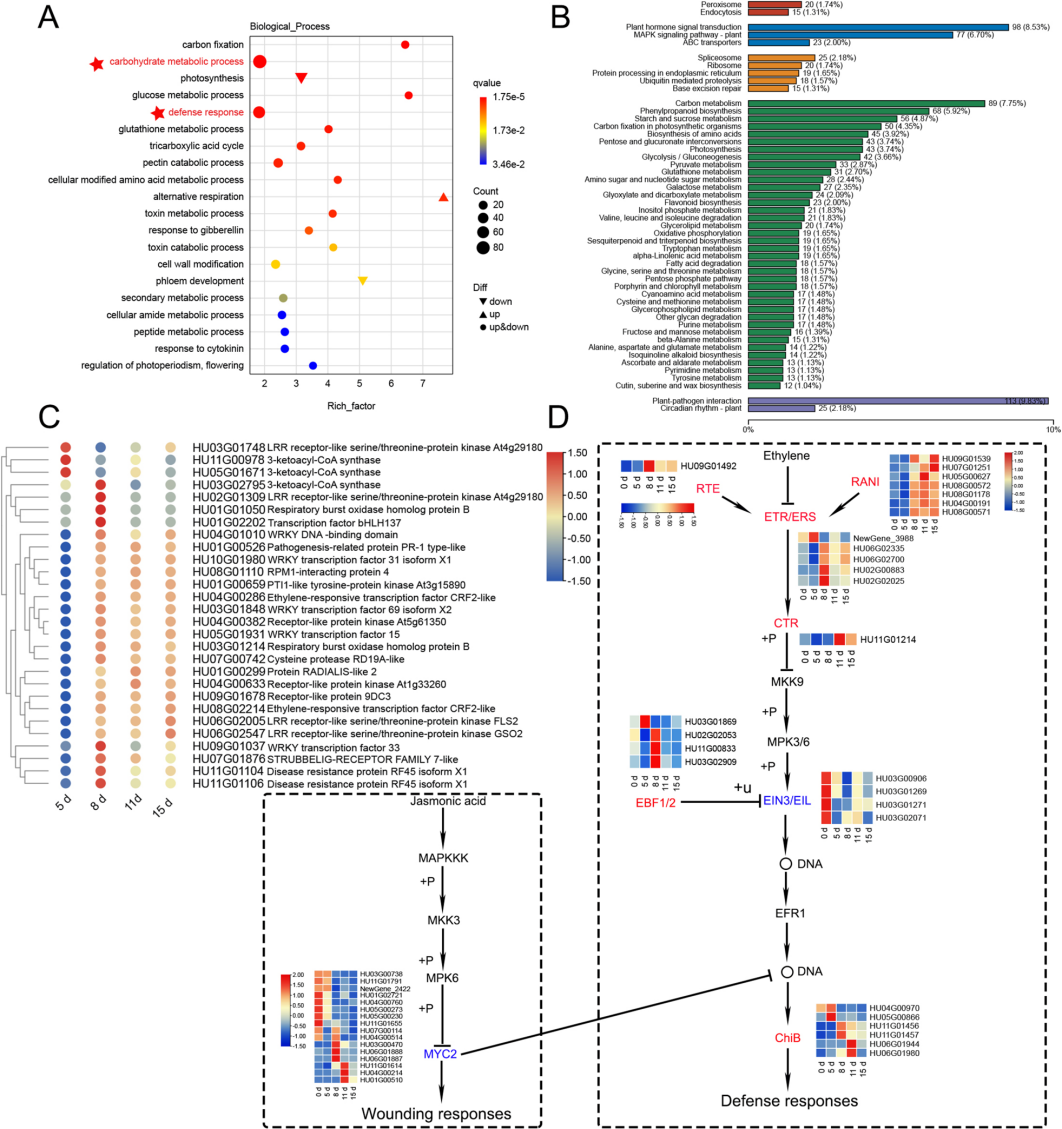
Identification and functional characterization of the differentially expressed genes at 8 days. **A** GO enrichment of pitaya infected with *N. dimidiatum*. **B** KEGG enrichment of pitaya infected with *N. dimidiatum*. **C** Heatmap analysis of plant-pathogen interaction genes when pitaya was infected with *N. dimidiatum* at different stages. **D** Expression profile and KEGG pathway analysis of ethylene and jasmonic acid-related genes involved in pathogen defense. (The KEGG pathway that the image comes from MAPK signaling pathway—plant (ko04016). Permission to use and adapt this image has been granted by Kanehisa Laboratories.)

We analyzed the DEGs for 8 days and found that defense response and carbohydrate processes were significantly enriched (Fig. 4A, B). Heatmap analysis and annotation of the enriched phytopathogenic interaction genes showed that the transcription factors *bHLH*, *WRKY* family, and Respiratory burst oxidase homolog proteins B (RBOHB) were up-regulated (Fig. 4C). In plants, ROS produced by the expression of respiratory burst oxidase homolog (Rboh) play a crucial role in regulating stress responses and enhancing resistance to pests and diseases [18]. Rbohs can bind to ROS production through calcium signaling and protein phosphorylation, and they are positioned at the center of the ROS cell network. The transcription factor *bHLH* acts as a molecular switch to control Rboh-dependent mechanisms in plant responses to biotic and abiotic stresses [18]. In addition, 3-ketoacyl-CoA synthase, a crucial gene involved in the regulation of long-chain fatty acid biosynthesis, which is a precursor for the synthesis of plant cuticle and wax layer, was significantly up-regulated. Photosynthesis in plants showed a significant down-regulation of expression (Fig. 4C).

Phytohormone enrichment increased dramatically at 8 days KEGG (Fig. 4B), and gene annotation revealed a significant downregulation of proteins related to indole-3-acetic acid (IAA) (Table S3). In addition to protecting against pathogenic fungi, ethylene (ET) can also enhance tolerance to pathogens. We found that the negative regulators of ET signaling, ETR/ERS and ERF1, were upregulated, suggesting that the synthesis of ET was inhibited. Furthermore, we found that the negative regulator of JA, MYC2, was downregulated (Fig. 4D). MYC2 is an important regulator in the JA signaling pathway [19], controlling the transmission and response of JA signals. This suggests that the synthesis of JA is also suppressed. More importantly, the receptor for ABA, PYR/PYL, was highly induced to upregulate its expression (Table S3). ABA generally defends against pathogenic fungi by regulating the opening and closing of stomata [20]. This suggests that pitaya is an important method for inhibiting the invasion of pathogenic fungi by regulating stomata through ABA [21, 22].

### Expression modulation at 11 days

**Fig. 5:**
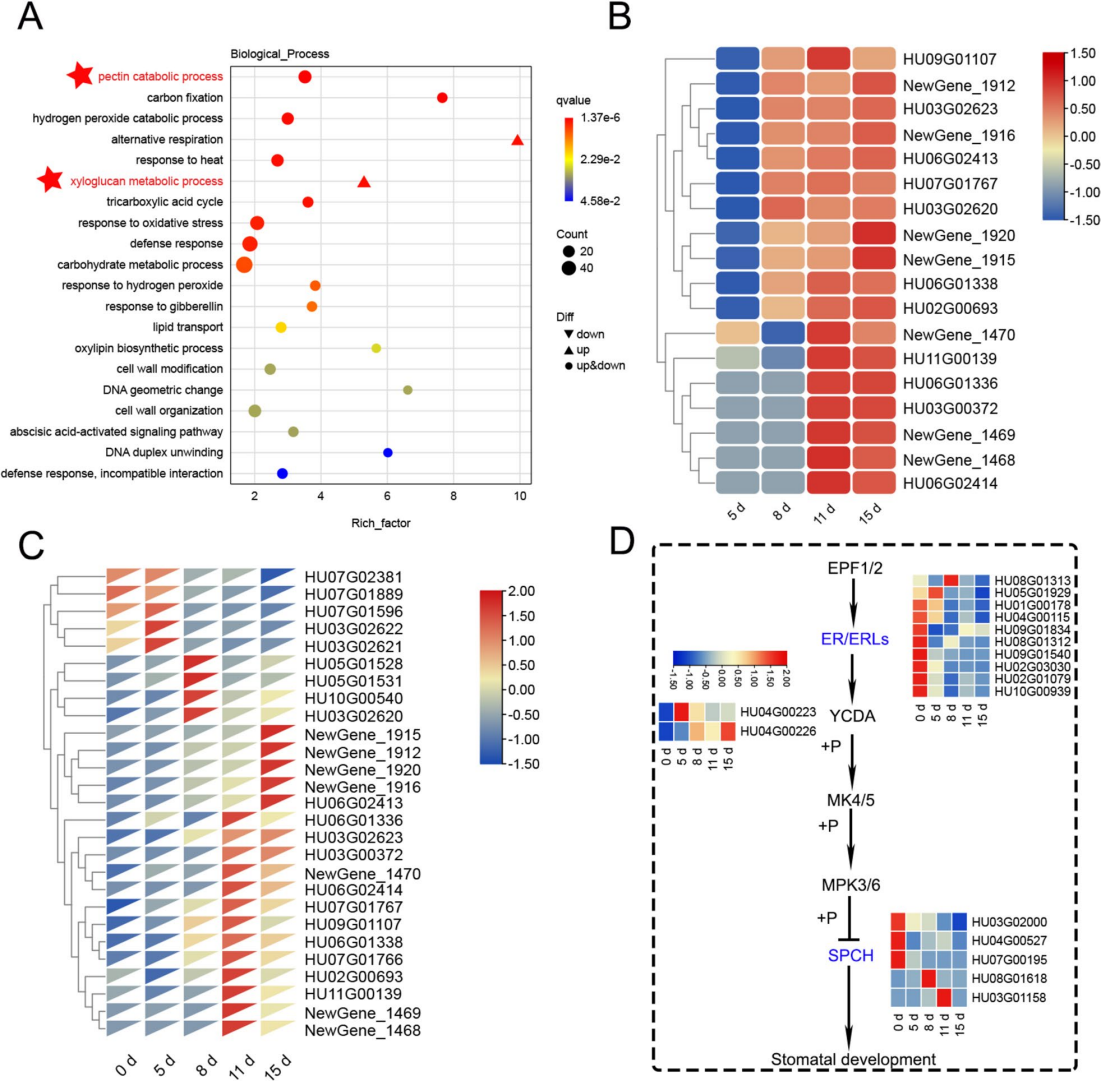
Identification and functional characterization of the DEGs at 11 days. **A** Go Enrichment analysis of pitaya DEGs at 11 days. **B** Heatmap of pectinase gene expression at different stages. **C** Heatmap of enrichment to xyloglucan endotransglucosylase gene expression at different stages. **D** Plant stomatal development-related gene expression and related pathways

We conducted Go enrichment analysis of pitaya at 11 days and observed a significant up-regulation of the Xyloglucan metabolic process (Fig. 5A, B). This process is a component of hemicellulose in the plant cell wall and serves as an inducer of active defense functions in the plant body [23]. We also observed a significant enrichment of the pectin catabolic process, with pectinases mainly involved in the pentose and glucuronate interconversions pathway. This may be because plant cells are being damaged by pathogenic fungi or undergoing programmed cell death (PCD) in pitaya. However, through the pentose and glucuronate interconversion pathway, pitaya can reengage in cell wall assembly and remodeling [24]. Additionally, pectinase can weaken the cell wall of the plant, increasing the degree of stomatal opening and maintaining the plant’s respiratory function [25]. Genes associated with pitaya stomatal development were significantly repressed in this pathway. This pathway is also involved in the pitaya defense pathway (ko04016), which can interact with RAR1 and SGT1 to induce hypersensitive response (HR) responses in plants (Fig. 5C, D).

### Expression modulation at 15 days

**Fig. 6:**
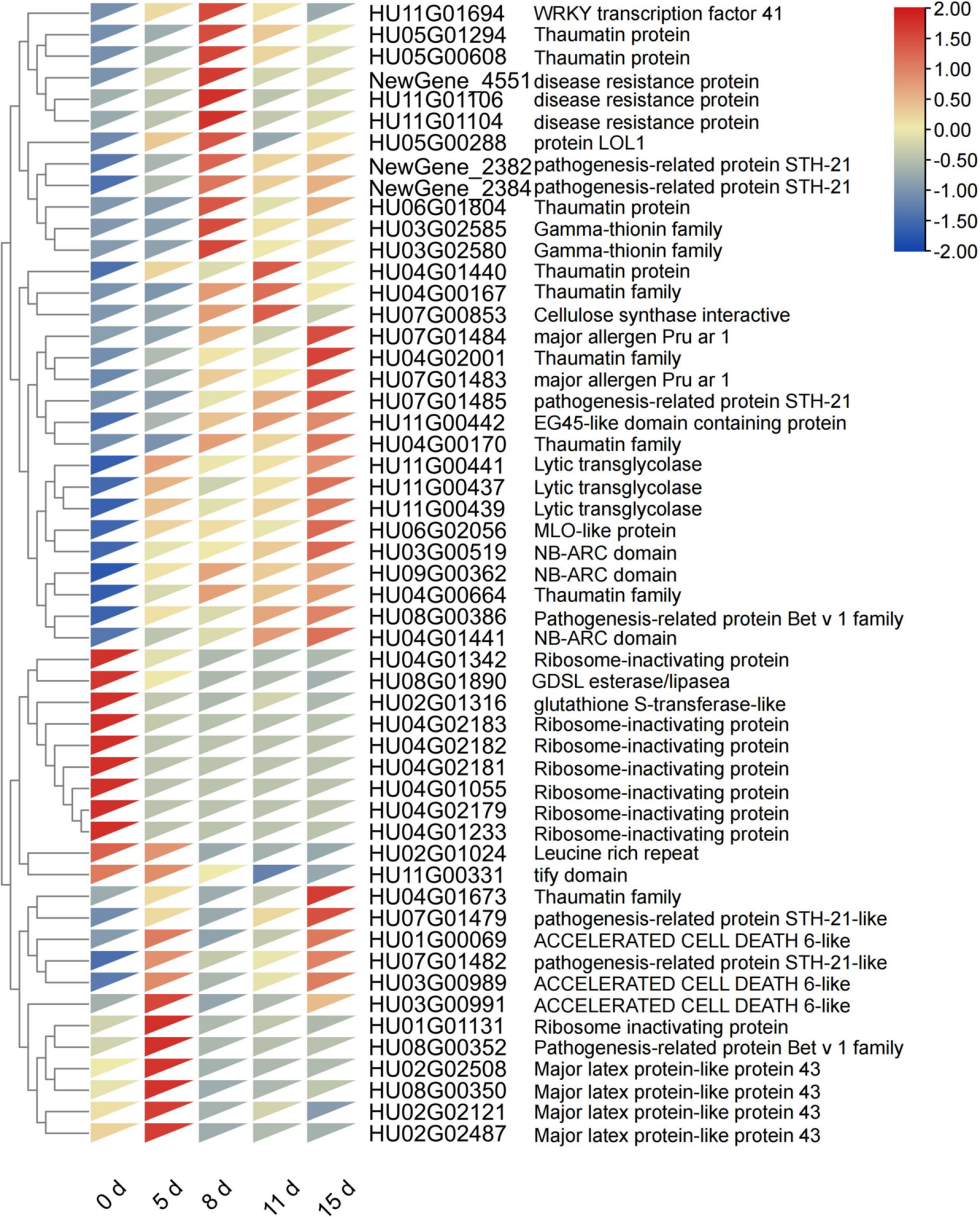
Heatman analysis of pitaya defense-related genes at different stages of pitaya infected by *N. dimidiatum*

We found significant enrichment in defense responses and carbohydrate metabolism during the RNA-seq data analysis of pitaya at 15 days. Heatmap analysis of defense-responsive genes showed a significant up-regulation of Thaumatin protein, diseases resistance protein, STH-21, Gamma-thionin, and other related resistance proteins (Fig. 6). Thaumatin protein inhibited pathogenic and non-pathogenic fungi by lysing fungal spores, inhibiting spore germination, and reducing the viability of young mycelium [26]. We also analyzed the enriched tricarboxylic acid cycle (TCA) and carbon fixation-related genes. The results showed that phosphoenolpyruvate carboxylase I and phosphoenolpyruvate carboxylase II were downregulated, which are key enzymes that play a crucial role in converting phosphoenolpyruvic acid (PEP) to oxaloacetic acid in the crassulacean acid metabolism (CAM) pathway, and the malate synthase gene was also downregulated in the TCA (Table S4). Additionally, we found that the SA signaling pathway is upregulated. This pathway is involved in the ko04016 pathway and can regulate SGT1 through its interaction with PRS2. It can also regulate *WRKY1/2* transcription factors that induce the production of defense-related genes, such as *PR* genes, thereby promoting resistance gene production or programmed cell death (Table S5).

### Co-expression trend analysis of DGEs in pitaya at different infection periods

Genes in organisms tend to operate in concert to form networks**.** Co-expression trend analysis of DGEs at 5 time points with k-means clustering revealed that the genes were classified into 15 modules (Fig. S4A). Clusters 2, 7, 9, and 14 exhibit trends in gene expression from the early to middle stages of infection, while clusters 3, 4, 8, and 13 display gene expression patterns during the middle to late stages. Clusters 10, 12, and 15 exhibit sustained gene expression. Cluster 9 shows high expression at day 5 and was enriched for proteins responsive to pathogenic fungi (Fig. S4B), including the Hsp90 family, which were induced and up-regulated. Clusters 2 and 7 were enriched with proteins responsive to pathogenic fungi (Fig. S4C, D), such as Gamma-thionin, Thaumatin-like proteins (TLPs), and PR-4 family. These genes play an important role in responding to and defense against pathogenic fungi. Contained genes related to ROS burst through the WAK receptor, such as the WRKY33 family, calmodulin-like protein, and RBOHs. The wall-associated receptor kinase is a receptor for pectin, and pectin fragmentation by external attack causes plant ROS burst [24]. In addition, cluster 14 was enriched for genes involved in the early plant defense response against pathogens, such as pectin methylesterase and xyloglucan endotransglucosylase (Fig. S4E).

The analysis of genes in clusters 3, 4, 8, and 13 revealed that these clusters were associated with photosynthesis, carbon fixation, TCA, and pathogenic defense (Fig. S4F, G, H, I). Phosphoenolpyruvate carboxylase-related genes were down-regulated in the TCA (Fig. S4F). Photosystem II core complex protein psbY, Photosystem I psaG/psaK, photosynthetic NDH subunit of subcomplex B, and other related genes were down-regulated in photosynthesis (Fig. S4G). The expression of the defense-related MLP-like protein was also down-regulated (Fig. S4I). Moreover, we observed a significant down-regulated expression of cluster 8 to JA, indicating an antagonistic role between JA and SA in the defense process of pitaya (Table S6, Fig. 4, Fig. S4H).

Genes in clusters 10, 12, and 15 were involved in various biological processes. For example, cluster 10 was enriched in genes related to plant waxy layer synthesis, such as cytochrome P450 and alcohol-forming fatty acyl-CoA reductase (Fig. S4J). The lipid metabolism-related gene, non-specific lipid-transfer protein, and phenylpropanoid biosynthesis were also enriched. Additionally, cluster 12 focuses on protein phosphorylation and defense response genes (Fig. S4K, Table S6), such as NTM1, accelerated cell death, etc. Furthermore, the regulation of ABA-related genes was enriched in cluster 15 (Fig. S4L), including STH-21 and lachrymatory-factor synthase. These findings indicate that after detecting the pathogenic fungus, the plant continuously slows or hinders further invasion through plant wax layer synthesis, cell wall synthesis, and the induction of SA and ABA production.

### WGCNA analysis

**Fig. 7:**
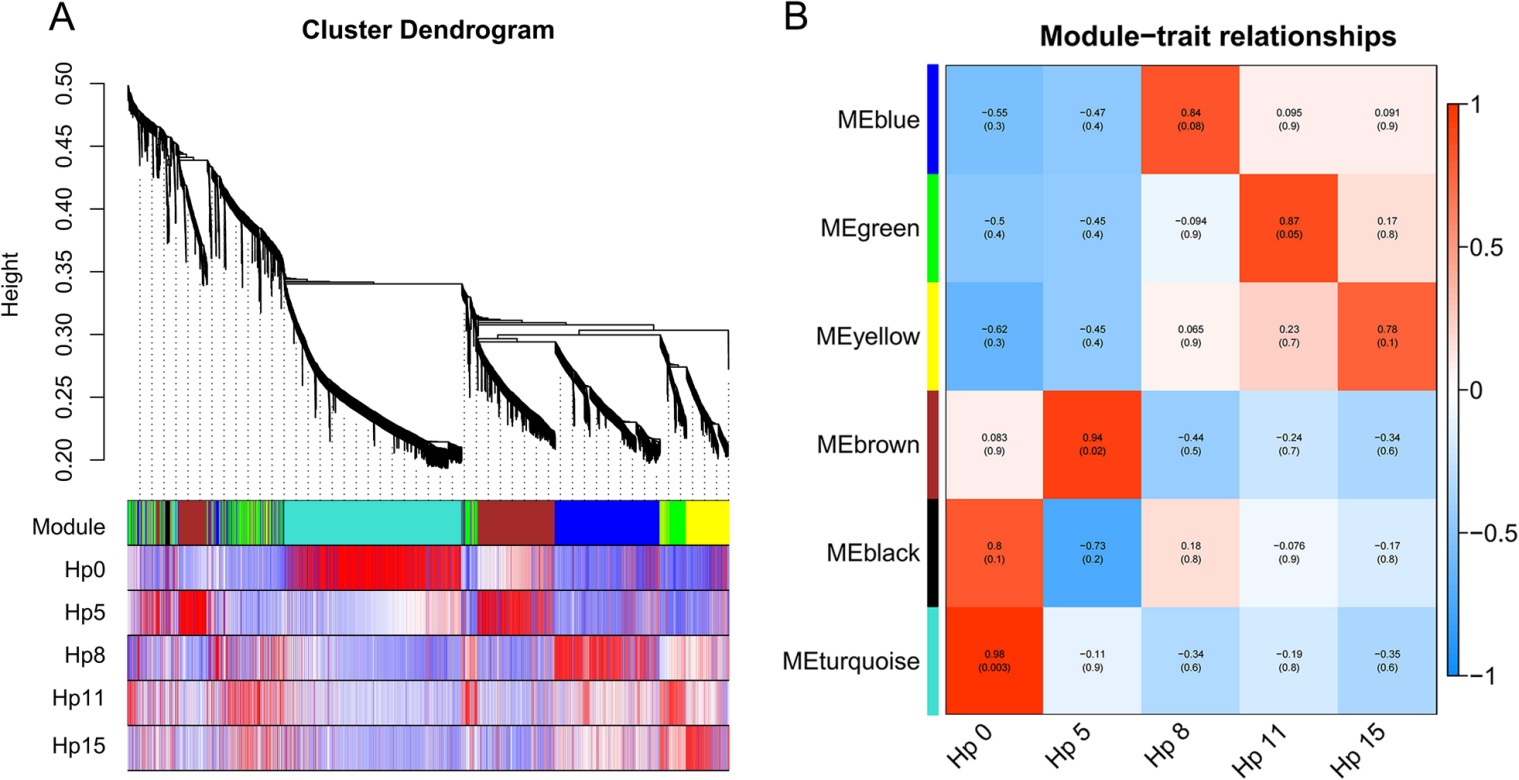
WGCNA analysis of pitaya at different interaction stages with *N. dimidiatum*. **A** Hierarchical cluster trees showing the co-expression modules identified by WGCNA. **B** WGCNA co-expression modules. Correlation of modules (left) and features (bottom). Red and blue represent positive and negative correlations, respectively

We used WGCNA to develop gene modules relevant to plant pathology. We screened for DEGs with FC ≥ 4 and identified genes using the dynamicTreeCut algorithm in R software, assigning a different color to each module. The hierarchical clustering tree is divided into five modules (Fig. 7A, B). Subsequently, we analyzed and explored these five modules.

The healthy pitaya branch (Hp 0) was associated with the turquoise module, which primarily focused on carbon fixation, TCA, photosynthesis, and all up-regulated expression of genes involved in maintaining plant growth (Fig. S5A). The infected pitaya branches (Hp 5, Hp 8) were associated with the brown and blue modules, respectively. The brown module mainly focused on the heat response and light response (Fig. S5B), whereas the blue module focused on mitochondrial cytochrome c oxidase assembly, cell wall macromolecule catabolic process, chitin catabolic process, response to SA, and defense response (Fig. S5C). The defense response genes mainly regulate the plant defense response through ERF1, which is a regulator of ET that acts on SGT1 to produce the HR response in plants.

**Fig. 8:**
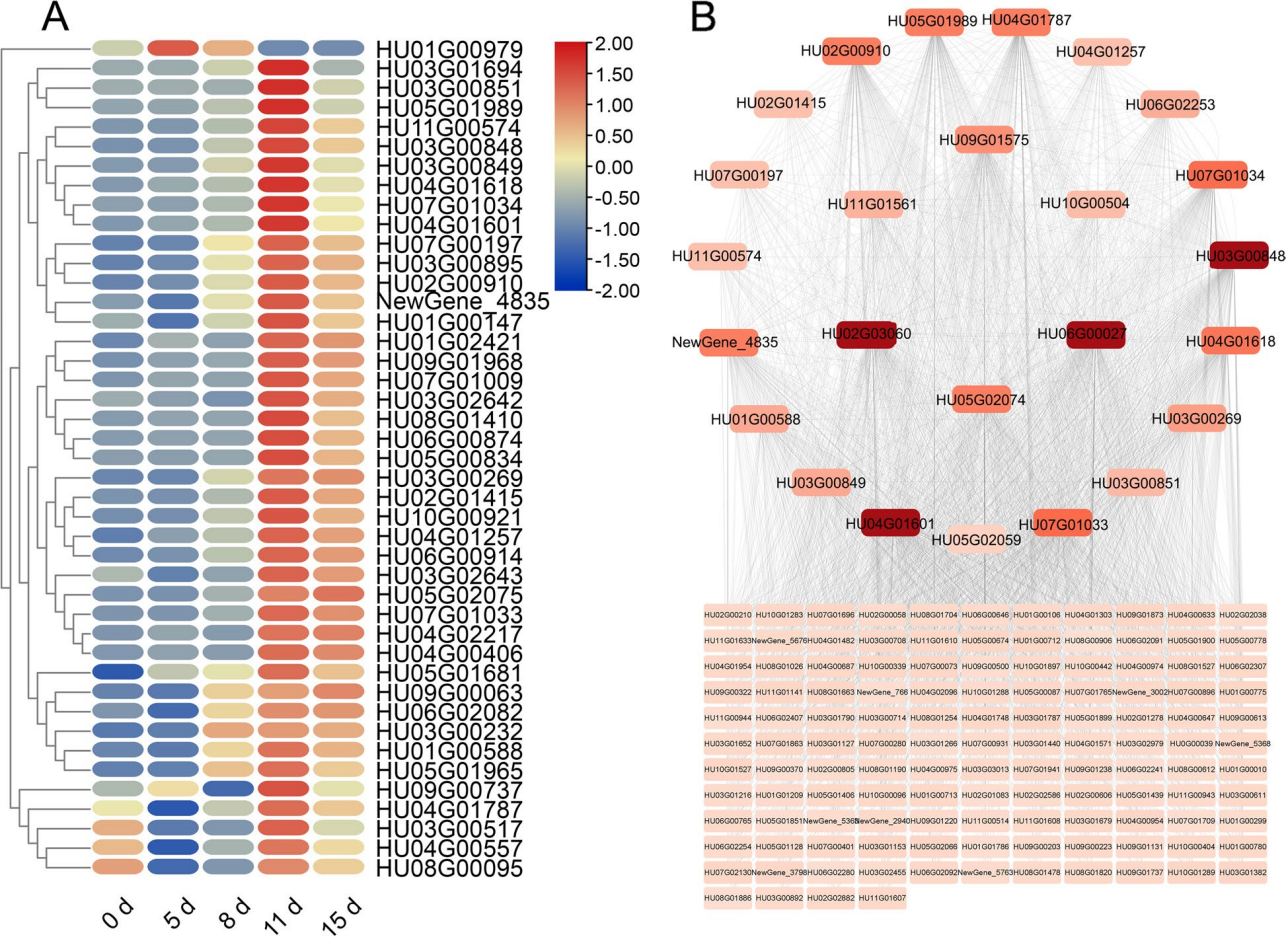
Expression heatmap and network of protein interactions of phenylpropanoid biosynthesis-related genes. **A** Heatmap analysis of 44 genes related to phenylpropanoid biosynthesis. **B** Network protein interaction map of genes related to phenylpropanoid biosynthesis and transcription factors

The infected pitaya branches (Hp 11 and Hp 15) were associated with the green and yellow modules, respectively. Notably, they were mainly focused on phenylpropanoid biosynthesis and flavonoid biosynthesis (Figure S5D, E). Phenylpropanoid biosynthesis can produce various metabolites, such as flavonoids, lignans, and cinnamic acid amides, etc., which can strengthen plant cell walls, scavenge reactive oxygen species, and protect DNA [27]. We conducted a heat map analysis of 44 genes associated with phenylpropanoid biosynthesis and discovered that they were significantly induced at 11 days. This suggests that plants activate defense mechanisms against pathogenic fungi by reinforcing the plant cell wall and repairing damaged plant tissues (Fig. 8A). We selected genes associated with phenylpropanoid biosynthesis and six transcription factors related to network protein interactions: *NAC (HU09G01575*, *HU02G03060*), *GATA (HU05G02074)*, *RAX3 (HU06G00027)*, *MYB* (*HU11G01561*), and *BZIP* (*HU02G01231*). These factors exhibited a strong correlation with each other (Fig. 8B).

### Changes in gene expression profiles of *N. dimidiatum* during pitaya infection by *N. dimidiatum*.

**Fig. 9:**
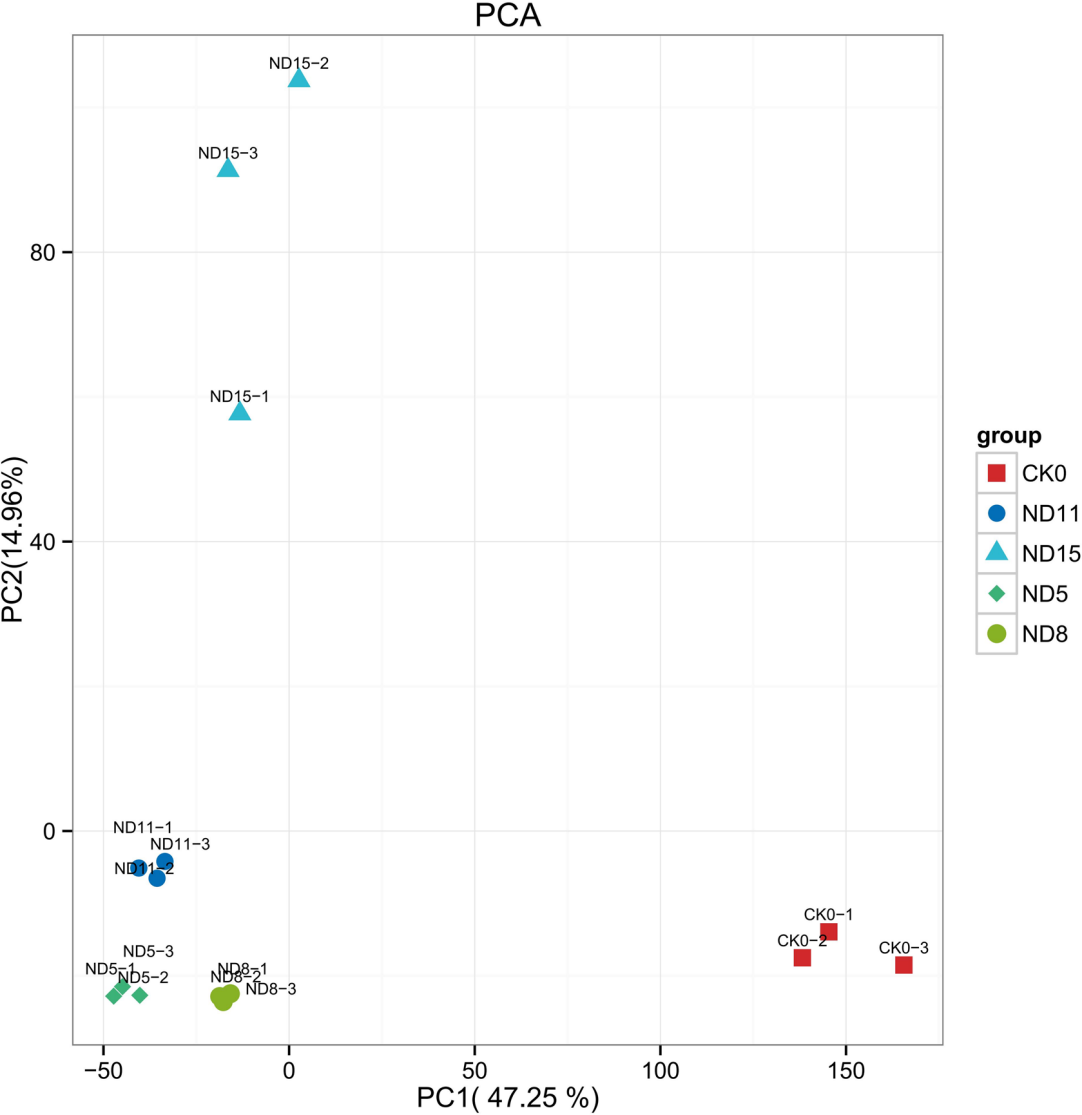
Principal component analysis (PCA) of *N. dimidiatum*

To verify changes in pathogen-specific gene expression during infection and understand the adaptive adjustment of genes under its virulence and environmental adaptation, we analyzed the entire transcriptional process of *N. dimidiatum* after infestation (Fig. 9).

### Expression of *N. dimidiatum* virulence factors

**Fig. 10:**
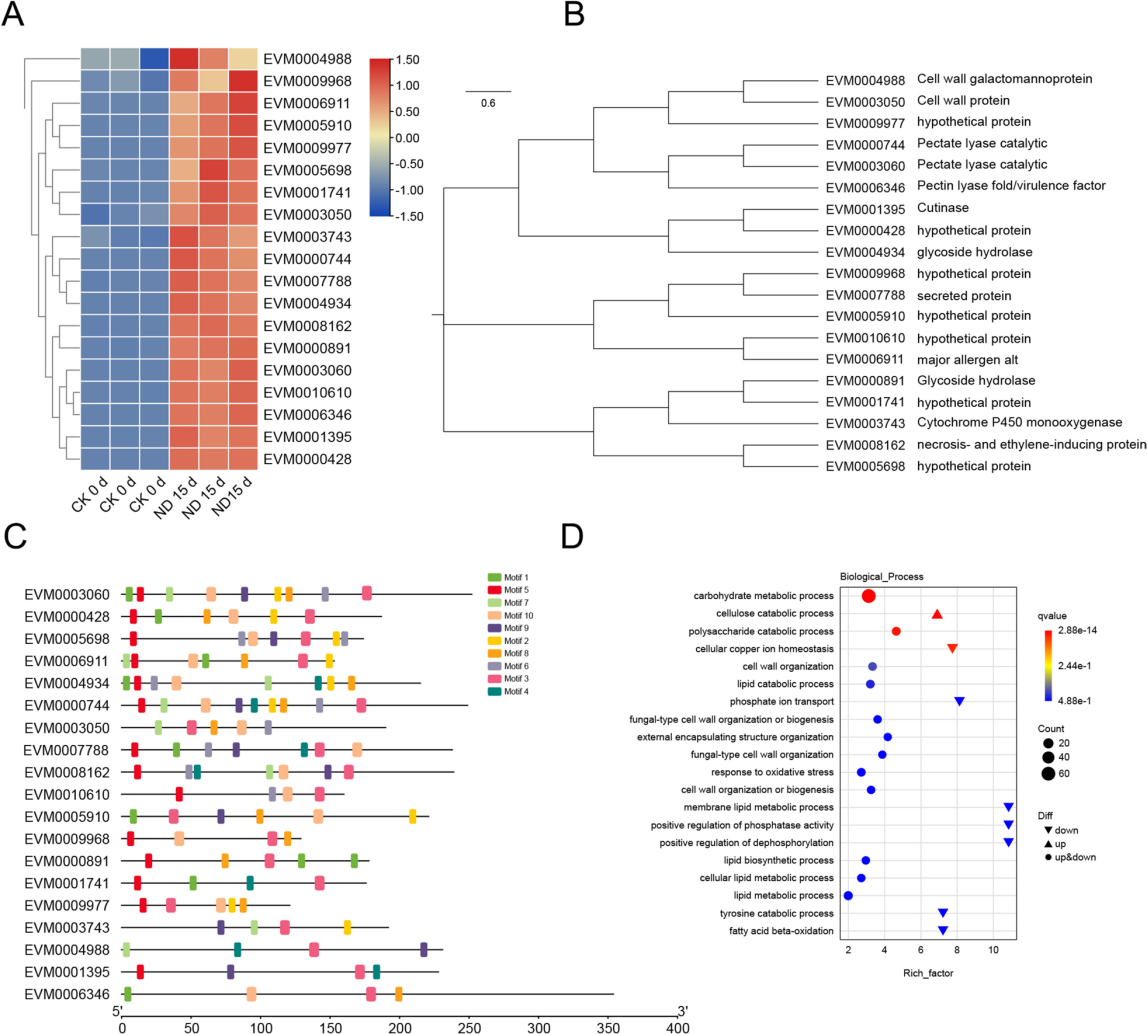
Gene expression and structural analysis of *N. dimidiatum* effector proteins. **A** Heat map analysis of the 19 effector proteins before and after infestation by *N. dimidiatum*. **B** Phylogenetic tree analysis of the 19 effector proteins. **C** Motif analysis of 19 effector proteins. **D** Go enrichment analysis of *N. dimidiatum* DEGs

Effector proteins of pathogenic fungi often play a crucial role in the successful invasion of plants and are an essential component of pathogen virulence [28]. We found that 19 genes, which matched with the genomically predicted effector proteins of *N. dimidiatum*, showed significantly upregulated expression (Fig. 10A). Their evolutionary relationships are shown in Fig. 10B. These genes are pectinase, carbohydrase, cutinase, and glycoside hydrolase, which are typical virulence factors. The phylogenetic tree analysis revealed that these virulence proteins were divided into different clusters, indicating that they evolved separately but cooperated in function. We predicted the motif structure for these genes and found that the 5' end was more conserved. Among these motifs, 3, 5, and 10 were more conserved and present in almost every gene (Fig. 10C). These conserved motifs often play a significant role in the recognition and virulence of pathogenic fungi during plant invasion.

**Fig. 11:**
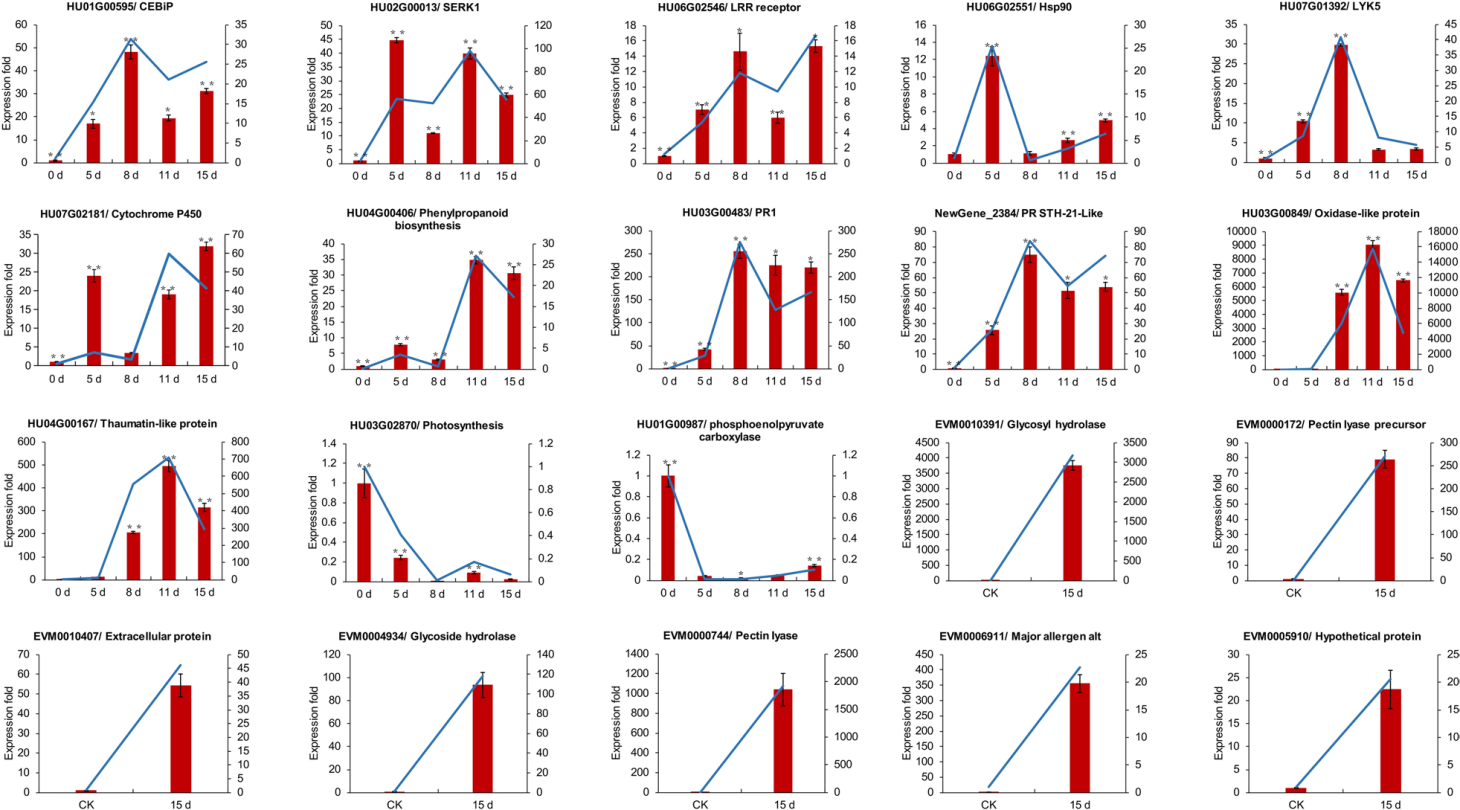
qRT-PCR to validate significantly differentially expressed genes. The red bar and the blue line graph represent the qRT-PCR and RNA-seq data, respectively. Data are presented as the mean ± standard error (SE). * represents a *p*-value < 0.05, ** represents a *p*-value less than 0.01

We found that carbohydrate metabolism and cellulose catabolic were significantly enriched by GO enrichment analysis, which could disrupt the cell wall of plants, and pectin, and break down these complexes into monosaccharides that can be absorbed by themselves (Fig. 10D). Pectin, in contrast, is the most variable, degradable, and dynamic polysaccharide in plant cell walls. It also plays a crucial role in detecting cell wall integrity. When pathogenic fungi disrupt the plant cell wall, they are recognized by receptors on the plant cell wall, initiating intracellular signal transduction and response mechanisms to impede pathogen invasion. We verified the expression of DEGs of *N. dimidiatum* and pitaya at different times by RT-qPCR (Fig. 11).

## Discussion

In this study, capturing the RNA gene expression profiles of both pitaya and *N. dimidiatum* during infection provided valuable insights into how pitaya recognizes *N. dimidiatum.* It also revealed the time point at which pitaya generates an immune response to *N. dimidiatum*. This information is crucial for understanding the dynamic changes in gene expression between pitaya and *N. dimidiatum*, as well as for more effective search for defense against *N. dimidiatum* targets.

At the plant cell surface, receptor-like kinases (RLKs) and receptor-like proteins (RLPs) function as pattern recognition receptors (PRRs) to perceive pathogen-associated molecular patterns (PAMPs). Plant PRRs can be subdivided into three categories based on the nature of their ligand-binding ectodomains: leucine-rich repeat (LRR), lysine motifs (LysM), and lectin [10]. At the 5 days of *N. dimidiatum* infection with pitaya, a large number of *N. dimidiatum* invaded the stomata of the pitaya (Fig. S1). Transcriptome data showed significant upregulation of genes containing the LRR structural domain, including *New_gene 3625*, *New_gene 3211*, *New_gene 3205*, and *HU01G00646*. These genes can serve as PRRs to recognize pathogen-associated molecular patterns and activate the PTI response in pitaya. Such as bacterial flagellin or elongation factor Tu (EF-Tu) [29]. Similarly, genes containing the LysM structural domain were significantly up-regulated, including *HU01G00595*. They bind carbohydrate-based ligands, such as fungal chitin or bacterial peptidoglycan, which in turn binds to chitin elicitor-binding protein (CEBiP) to form dynamic complexes that activate plant signaling immunity [30]. Additionally, it has been demonstrated that LysM-containing receptor-like kinase 5 (LYK5) displays a higher chitin-binding affinity than CERK1 [31]. In fact, we observed fungal hyphae invading pitaya through its stomata on the 4th day of the pitaya- *N. dimidiatum* interaction (Fig. S1), indicating that the PTI response in pitaya was activated earlier. Meanwhile, on the 5th day, the pitaya's stomata were already damaged, suggesting that *N. dimidiatum* inhibited the pitaya PTI. We detected the highest expression of LYK5 (*HU05G00005*, *HU07G01392*) at 8 days (Figs. 3 and 10). However, further verification of its function is required. A significant up-regulation of numerous Hsp90 genes occurred (Table S2), peaking at 5 days and gradually decreasing over time. Hsp90 is a co-chaperone of SGT1, which is required for the maturation of NLR73 in various plant species and triggers the ETI response in plants [13]. Therefore, it can be used as a marker to monitor early infection of pitaya by *N. dimidiatum*.

A direct link between the activation of the PRR complex and ROS production can activate plant immune signaling, eventually leading to a series of powerful immune responses [32]. These responses include cell wall strengthening [33], protein inhibitors [34], peroxisomes, antifungal molecules [35], R proteins, silencing mRNA, and regulation of the plant-pathogen microbiota [13]. RBOHB (*HUO1G01050*, *HU03G01214*) and Ca^2+^ (*HU08G01602*, *HU06G01765*) were significantly up-regulated at 8 days, suggesting that the activation of the PRRs complex around 5 days triggers a local and systemic defense response. This response is accompanied by changes in Ca^2+^ levels, the production of ROS, and ongoing signaling to the nucleus. In plant nuclei, immune-related transcription factors bind to DNA and encode numerous antimicrobial genes, including *PR* genes, antifungal peptides, and thaumatin proteins, to inhibit the growth of pathogenic fungi. Our data also confirms the up-regulated expression of transcription factors *WRKY31* (*HU10G01980*), *WRKY69* (*HU03G01848*), *WRKY15* (*HU05G01931*), and *WRKY33* (*HU09G01037*), among others, which play a role in the plant's systemic defense against pathogenic fungi. In Arabidopsis, studies have shown that *WRKY33* is responsible for PAMP-induced antimicrobial toxin production [36]. In addition, we found that photosynthesis and phosphoenolpyruvate carboxylase synthesis were significantly inhibited at 8 days and peaked at 15 days. This suggests a possible link to *N. dimidiatum* attack on pitaya chloroplasts.

In addition to immune receptors, phytohormones may regulate immune signaling by controlling the basal levels of signaling components in cells [37]. The expression of pitaya disease resistance-related hormones, such as ET, JA, and ABA were also regulated. In studies, it was found that the synthesis of ET and JA in pitaya's defense response is inhibited. Previous research has shown that pathogenic fungi can secrete toxins or inhibitors to interfere with plant growth and development, thereby inhibiting ET production. For example, *PsAvh238* interacts with Type2 ACSs (GmACSs), disrupting their stability and inhibiting ET biosynthesis, which promotes infection by *P. sojae* [38]. This is consistent with the notion that ET-mediated defenses have to be downregulated in this hemibiotrophic fungi [39]. *MYC2* is an important switch in the JA-induced signaling pathway, playing a positive role in regulating the expression of genes associated with disease progression and mechanical damage. This enables plants to mount an effective defense against pathogenic fungi. Pathogenic fungi can inhibit the synthesis of MYC2 by secreting small molecular proteins, thereby interfering with the JA defense pathway and further invading pitaya [19]. On the contrary, SA and ABA were up-regulated at 8 days, suggesting that ABA could enhance the resistance of pitaya to *N. dimidiatum*. It is well known that SA induces transcriptional reprogramming, including the expression of pathogenicity-related (*PG*) genes, which inhibits the growth and spread of pathogens [39]. ABA is involved in regulating plant defense against various fungal pathogens. For example, the transcription factor *LeJA2* in tomato upregulates the expression of *LeNCED1*, promoting ABA synthesis, thereby restricting pathogen entry through stomata [40]. This finding is consistent with our observation of *N. dimidiatum* entering pitaya through stomata on the surface of pitaya [9]. In addition, ABA has been shown to improve disease resistance in Arabidopsis against the *Pseudomonas syringae* DC3000 strain [41]. However, plant hormones often form complex networks to collectively defend against pathogen invasion. How the synergistic effect between different hormones in pitaya effectively resists *N. dimidiatum* invasion needs further verification.

Phenylpropanoid and flavonoid biosynthesis enhance plant immunity and play an important role in plant defense responses [42]. Flavonoids are an important downstream branch of phenylpropanoid metabolism [42]. Peroxidase (*HU05G02059*, *HU01G00979*), caffeic acid 3-O-methyltransferase (*HU07G00197*, *HU06G00874*, *NewGene_4835*), and cytochrome P450 (*HU05G02075*) are up-regulated and expressed on 11–15 days. These genes ultimately contribute to the formation of lignin, which thickens the cell walls of plants and plays a crucial role in protecting against biotic and abiotic factors. In addition, the transcription factors *NAC* (*HU09G01575*, *HU02G03060*), *GATA* (*HU05G02074*), *RAX3* (*HU06G00027*), *MYB* (*HU11G01561*), and *BZIP* (*HU02G01231*) are involved in phenylpropanoid biosynthesis. We found that the biosynthesis of phenylpropanoids and flavonoids only began to respond at the late stage of *N. dimidiatum* infestation. This could be due to the pitaya's ability to enhance the formation of healthy lignin around the pathogen infestation, thereby inhibiting the continuous infestation by *N. dimidiatum*.

Adaptogenic pathogens have evolved numerous effectors to suppress host immunity and manipulate host metabolism to produce virulence that promotes plant susceptibility [13]. Pectinases (*EVM000744*, *EVM003060*), keratinases (*EVM001395*), glycoside hydrolases (*EVM004934*), and necrotic and ethylene-inducible proteins (*EVM008162*) appeared to be up-regulated in expression. These proteins are typical virulence factors that have already been demonstrated in *U. virens* and *Phytophthora* [43, 44]. Our next step will be to investigate the function of these individual genes in the infestation of pitaya by *N. dimidiatum*.

**Fig. 12:**
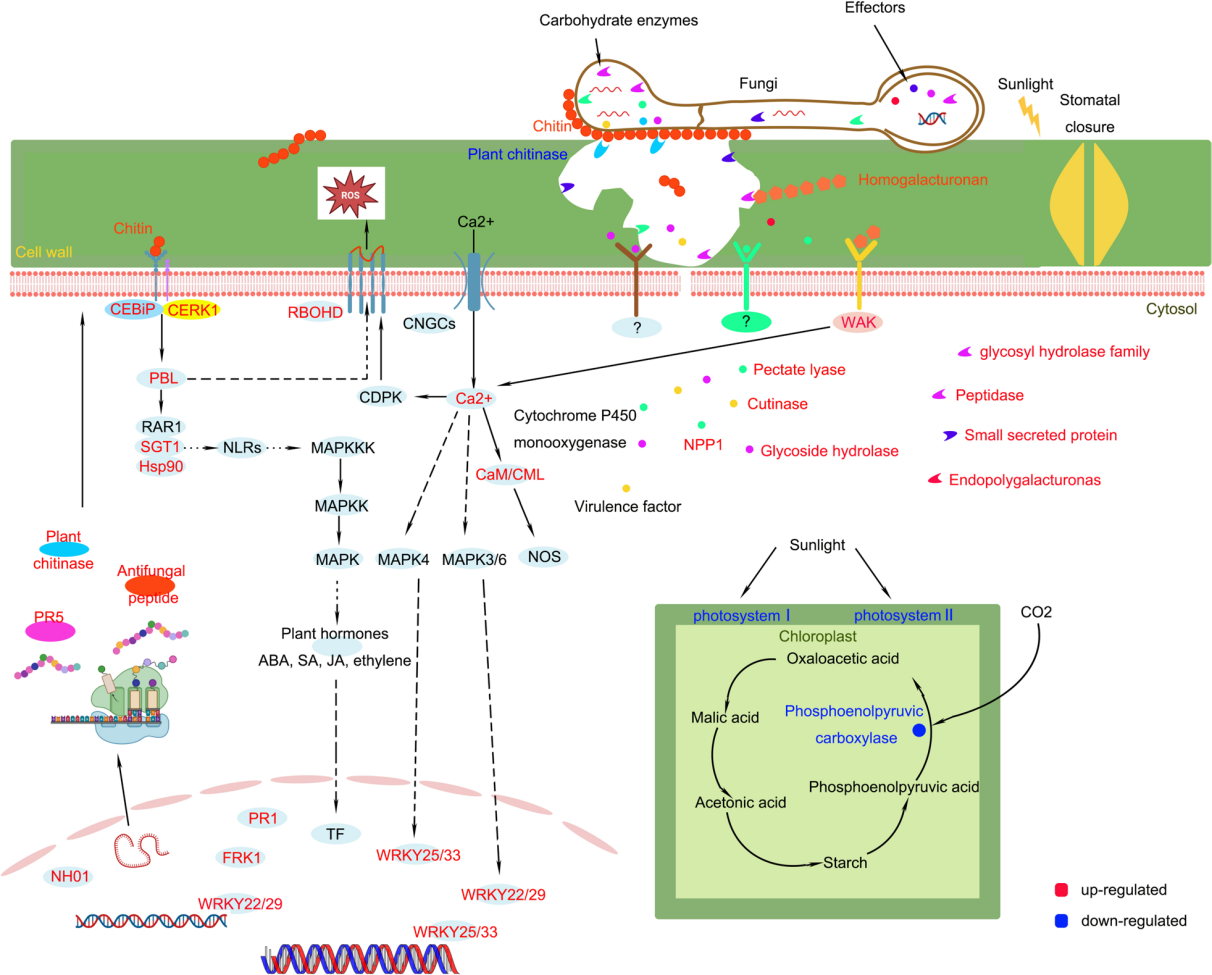
Gene pathways associated with the interaction between pitaya and *N. dimidiatum*. Red indicates up-regulated expression, and green indicates down-regulated expression. The solid arrows indicate the direct role, and the dashed arrows indicate the profile role

In summary, pitaya utilizes early immune receptors, amplification of immune signals, cellular reprogramming, phytohormones, and synthesis of phytohormones to prevent invasion by *N. dimidiatum* (Fig. 12). If the climate is suitable for the growth of *N. dimidiatum* during spring and autumn, it will rapidly invade the pitaya, leading to the destruction of pitaya cells. Conversely, in a severe environment, the pitaya exhibits a rapid defense response that can prevent the infiltration of pathogens. When combined with environmental constraints and nutritional stress, this defense mechanism can lead to the formation of spores by the pathogen. These spores can subsequently lead to re-infestation at a later when the stage environment improves. These studies demonstrate the effectiveness of combining the obtained RNA profiles with pathogenesis analysis for controlling pitaya canker. By controlling the secretion of key enzymes by *N. dimidiatum* or enhancing pitaya's ability to detect pathogens earlier, we can identify the critical processes that regulate pathogen colonization and infestation. This will provide opportunities to implement effective interventions.

## Materials and methods

Plant treatment: Healthy and uniformly grown old stems of pitaya "Jindu No. 1" were collected from Enhong Agricultural Technology Co., Ltd (18° 794 'N, 108° 688' E) in Banqiao Town, Hainan Province. The stems were cut to a length of about 20 cm for cultivation, and new shoots were allowed to grow to about 15–20 cm before conducting pathogen infection experiments. *N. dimidiatum* treatment: *N. dimidiatum* was cultured in PDA medium for 10 days, washed with ddH_2_O and filtered using triple-cleaning tissue paper. The concentration of spores was adjusted to 1 × 10^5^. The spores were evenly sprayed on the surface of pitaya while being kept moist, with three replicates for each sample. Pitaya stems were sprayed with sterile water as a control. Pitaya samples were taken and photographed at 5, 8, 11, and 15 days after spraying with *N. dimidiatum* spores.

### RNA isolation

The collected samples were ground into a powder using liquid nitrogen. Then, 0.1 g of the powder was weighed and transferred into a 2.0 mL EP tube. Next, 1.5 mL of a 2% CTAB lysis solution was added, followed by 2% β-mercaptoethanol. The sample was preheated at 65 °C for 30 min, then centrifuged at 12,000 × g for 5 min at 4 °C. The supernatant was pipetted into a 2.0 mL EP tube. Then, 200 μL of chloroform: isoamyl alcohol (24:1) was added per 1 mL of lysate. The mixture was mixed well and centrifuged at 12,000 × g for 10 min at 4 °C. Repeat the previous step by pipetting the supernatant into a new 1.5 mL centrifuge tube. Be cautious to avoid pipetting into the intermediate protein layer. Then, add 2/3 of the supernatant volume of isopropanol and mix well. Place the mixture at -20 °C for at least 2 h. Remove the supernatant and add 1 mL of 75% ethanol. Use a pipette to blow the precipitate and then dry it for 3–5 min. Dissolve the precipitate in 40 μL of DEPC water. The RNA concentrations and quality were verified using an Agilent 2100 Bioanalyzer (Agilent Technologies, Santa Clara, CA, USA).

### cDNA library generation and RNA-seq

Treatment of total RNA using mRNA enrichment and rRNA removal methods. Specifically, mRNA enrichment involves using magnetic beads with Oligo dT to enrich mRNA with a poly A tail. For rRNA removal, rRNA is hybridized with a DNA probe. RNase H selectively digests the DNA/RNA hybrid strand, while DNase I digests the DNA probe. Finally, the RNA is obtained after purification. The RNA was fragmented using a fragmentation buffer, reverse transcribed with random N6 primers, and synthesized into double-stranded DNA. The DNA was then filled and phosphorylated at the 5' end, with a protruding "A" sticky end at the 3' end. Finally, a complementary "T" nucleotide was added at the 3' end. The ligated product is amplified by PCR using specific primers. The PCR product is heat denatured to obtain a single strand. The cDNA library is created by cyclizing the cDNA with a bridge primer and then sequenced using a sequencing machine.

### Quality assessment of RNA-seq data

First, we use SOAPnuke [45] to filter out low-quality reads, reads contaminated with adapters, and reads with a high number of base N. The resulting filtered data is referred to as clean reads. We used HISAT2 [46] to align the clean reads to the reference genome sequence. Then, we used Bowtie2 [47] to align the clean reads to the reference gene sequence in order to obtain alignment results and identify new transcripts and novel genes. We add the newly identified transcripts with protein-coding potential to the existing reference gene sequence to create a comprehensive reference sequence. Subsequently, we calculate the expression levels of both genes and transcripts using RSEM [48]. Finally, we detect differentially expressed genes between different samples based on specific criteria. We then conduct in-depth clustering analysis and functional enrichment analysis of these differentially expressed genes, among other analyses.

### Trend analyses of DEGs

The differentially expressed genes (DEGs) were sorted according to different treatments and analyzed using the STEM software [49]. The parameters were set as follows: The maximum number of output profiles is 20, with similar profiles being merged. The minimum fold change ratio for DEGs is set at 2.0. The clustered profiles with a false discovery rate (FDR) ≤ 0.05 were considered significant profiles. Then, the DEGs in each profile or all profiles were analyzed for enrichment in Gene Ontology (GO) and Kyoto Encyclopedia of Genes and Genomes (KEGG) pathways.

### WGCNA analysis

Weighted gene co-expression network analysis (WGCNA) is a major method for constructing gene co-expression networks [50]. We utilized the R package WGCNA to analyze RNA-seq data and build gene co-expression networks. We estimated the Pearson correlation coefficient between genes based on their FPKM values by converting the correlation matrix into an adjacency matrix. Hierarchical clustering and the dynamic tree cut function were used to detect modules, grouping all genes into clusters. Different branches of the clustering tree represent various gene modules, where genes within the same module exhibit high co-expression levels, while genes belonging to different modules display low co-expression levels. The parameters were set with a minimum of 50 genes and a sensitivity of 3.0. Gene significance (GS) and module membership (MM) were calculated, and the information of the corresponding module genes was extracted for GO and KEGG analysis. Cytoscape was used for the visualization and analysis of the component genes.

### Quantitative real-time RT-PCR

Fluorescent quantitative polymerase chain reaction (RT-qPCR) was performed using SYBR® Premix Ex Taq. The reaction system consisted of 1 µL of cDNA (10 ×), 0.5 µL of upstream and downstream primers (final concentration of 10 μmol/L), and 10 µL of buffer, supplemented with water to 20 µL. Primers for the target genes were designed using Primer Premier 5 and are listed in Table S7. Three technical replicates and three biological replicates were available for each sample. Data analysis was performed using the 2^−ΔΔCt^ method. The *ubiquitin* gene of pitaya [51] and the *tubulin* gene of *N. dimidiatum* [5] served as the internal reference genes. SPSS v19.0 (SPSS, Chicago, IL, USA) was used to conduct a one-way analysis of variance (ANOVA) with Duncan’s multiple range post hoc test, and there was a significance threshold of *p* < 0.05.

### Conserved sequence analysis of genes and construction of evolutionary trees

The gene sequences were uploaded to the MEME online website (*MEME—MEME Suite (meme-suite.org*)) for analysis and the results were visualized using TBtools [52]. Phylogenetic tree analysis was performed using the Neighbor Joining (NJ) method with 1000 bootstrap replicates using MEGA 6.0 software [53].

### Supplementary Information

**Additional file 1: Supplementary Figure S1.** The dynamics of pitaya infection by *N. dimidiatum* were observed at different time points using microscopy. **Supplementary Figure S2.** The symptoms of pitaya plants were observed after being sprayed with *N. dimidiatum* spore suspension at 5, 8, 11, and 15 days. **Supplementary Figure S3.** Reproducibility between RNA-seq data replicates. **Supplementary Figure S4.** Co-expression trend analysis. **Supplementary Figure S5.** WGCNA divides DEGs into 5 modules, including Go enrichment analysis and heatmap analysis.

**Additional file 2:** **Table S1.** Total cDNA reads were mapped to the *N. dimidiatum* and pitaya using Bowtie2.

**Additional file 3: Table S2.** 5 d fold protein annotation.

**Additional file 4:** **Table S3.** 8 d IAA gene expression and annotatin analysis.

**Additional file 5: Table S4.** 15 d carbon fixation and tricarboxylic acid cycle gene expression and annotatin analysis.

**Additional file 6: Table S5.** Salicylic acid gene expression and annotatin analysis.

**Additional file 7: Table S6.** Protein phosphorylation gene expression and annotatin analysis.

**Additional file 8: Table S7.** Primer sequences were used for this study.

## Data Availability

The original contributions presented in the study are included in the article/Supplementary material. RNA-seq datasets are available in the NCBI under accession number PRJNA1027117.

## Abbreviations

DEG: Differentially Expressed Genes
GO: Gene Ontology
KEGG: Kyoto Encyclopedia of Genes and Genomes
*PR* gene: Pathogenesis-Related gene
qPCR: Quantitative Real-time PCR
PRRs: Pattern Recognition Receptors
PAMPs: Pathogen-Associated Molecular Patterns
PTI: Pattern-Triggered Immunity
NLR: Nucleotide-binding domains and Leucine-rich Repeat
ETI: Effector-Triggered Immunity
dpi: Days post-inoculation
HR: Hypersensitivity reactions
RBOHs: Respiratory Burst Oxidase Homolog Proteins B
IAA: Indole-Acetic Acid
SA: Salicylic Acid
JA: Jasmonic acid
ABA: Abscisic Acid
PCD: Programmed Cell Death
TCA: Tricarboxylic Acid Cycle
