# Supplementary material for: Host-pathogen interaction between pitaya and Neoscytalidium dimidiatum reveals the mechanisms of immune response associated with defense regulators and metabolic pathways
Source: BMC Plant Biol. 2024 Jan 2;24:4. doi: 10.1186/s12870-023-04685-y (PMC10759344; doi:10.1186/s12870-023-04685-y)
Supplement: Supplementary file 1 — Additional file 1: Supplementary Figure S1. The dynamics of pitaya infection by N. dimidiatum were observed at different time points using microscopy. Supplementary Figure S2. The symptoms of pitaya plants were observed after being sprayed with N. dimidiatum spore suspension at 5, 8, 11, and 15 days. Supplementary Figure S3. Reproducibility between RNA-seq data replicates. Supplementary Figure S4. Co-expression trend analysis. Supplementary Figure S5. WGCNA divides DEGs into 5 modules, including Go enrichment analysis and heatmap analysis. [file 12870_2023_4685_MOESM1_ESM.docx]

**
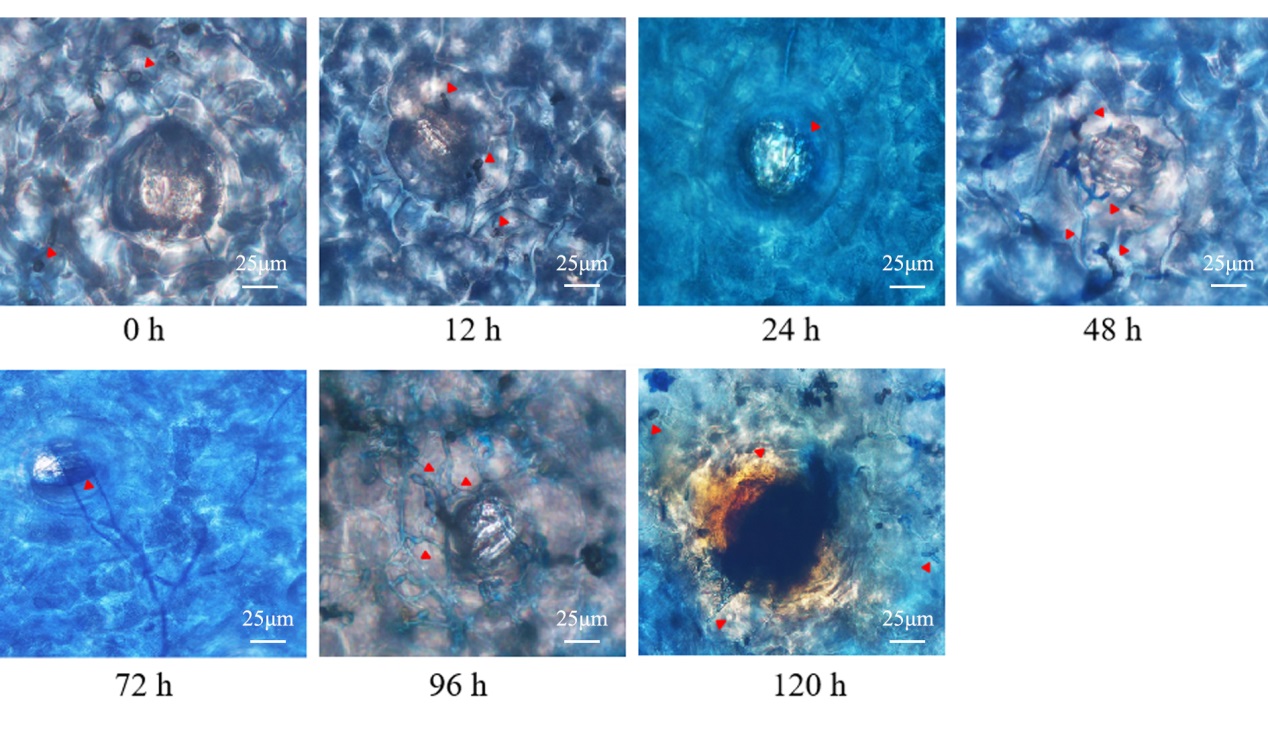
****Supplementary Figure S1.** The dynamics of pitaya infection by *N. dimidiatum* were observed at different time points using microscopy. The mycelium of *N. dimidiatum* could invade pitaya through stomata. The red triangles represent spores and mycelium at various stages.


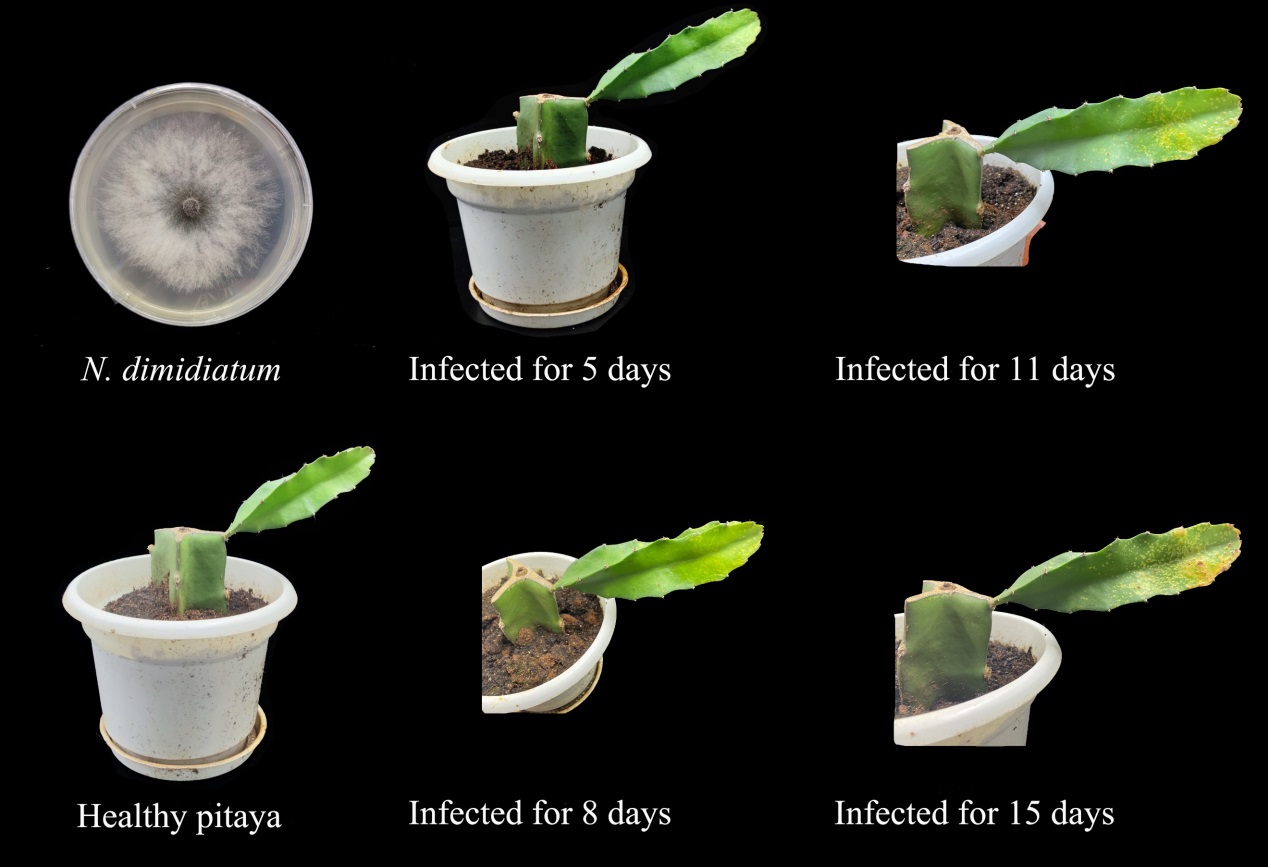


**Supplementary Figure S2.** The symptoms of pitaya plants were observed after being sprayed with *N. dimidiatum* spore suspension at 5, 8, 11, and 15 days.
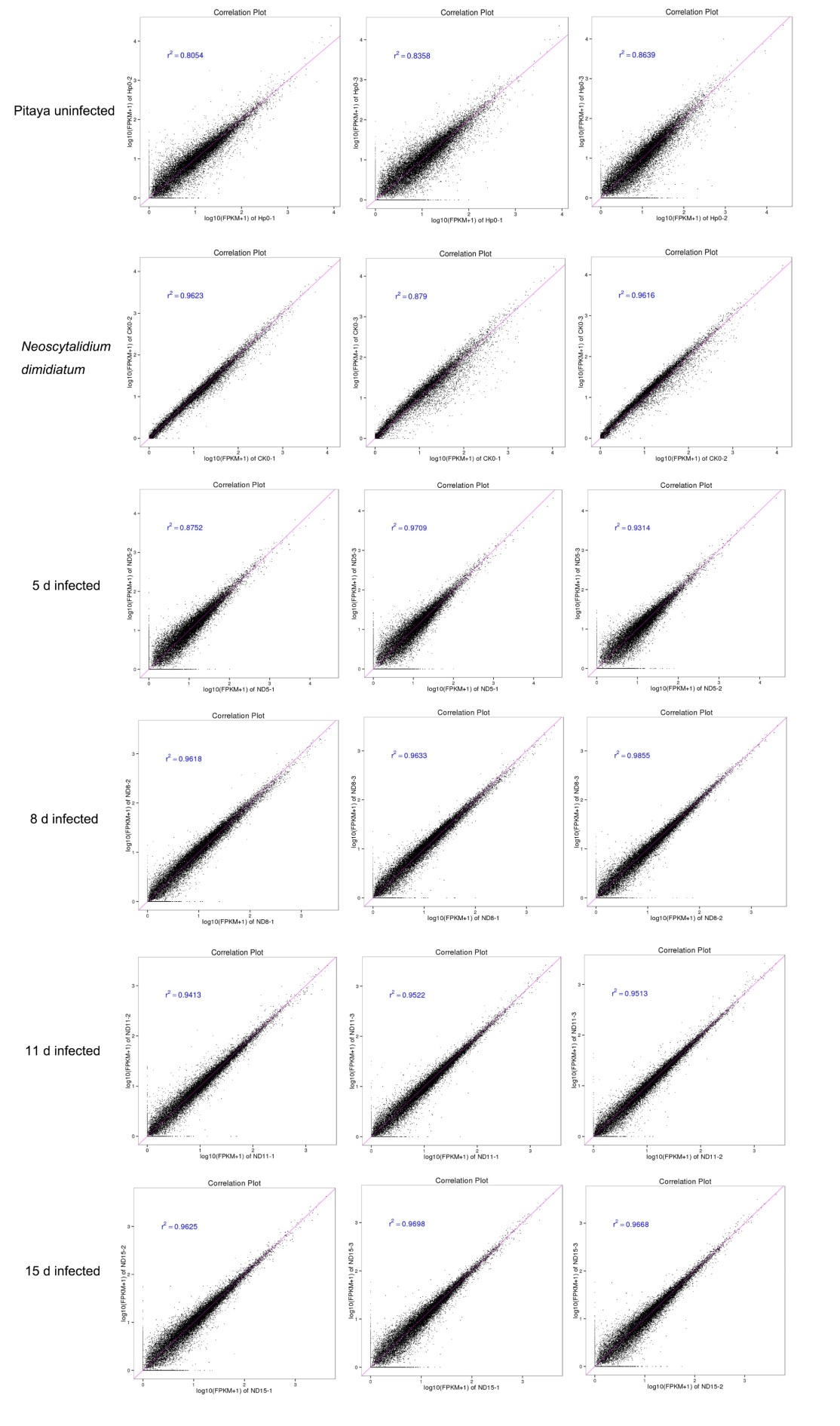


**Supplementary Figure S3.** Reproducibility between RNA-seq data replicates. RPKM normalized read counts for all detected. Pitaya or *N. dimidiatum* genes are plotted for 3 biological replicates. The Pearson correlation coefficient (r) is given for each replicate.


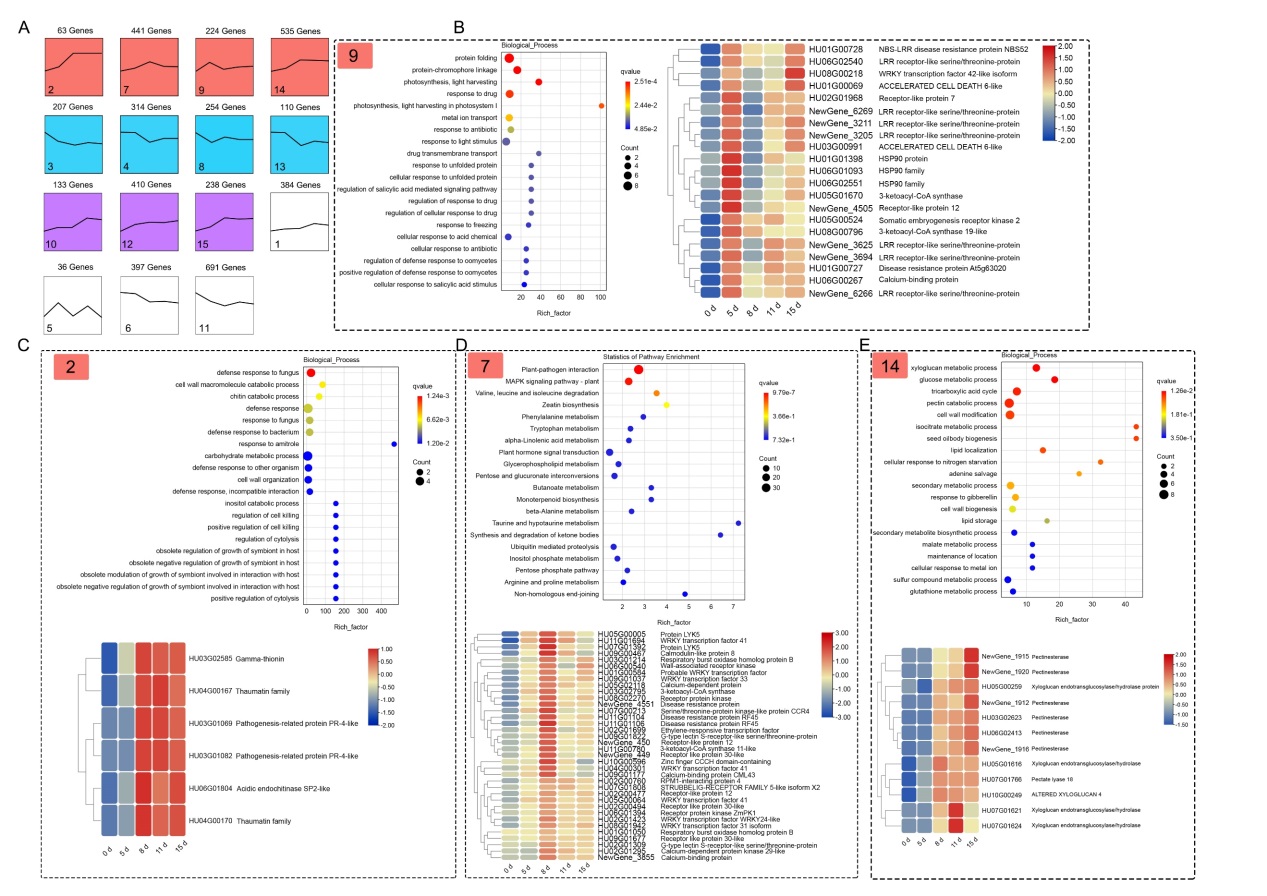


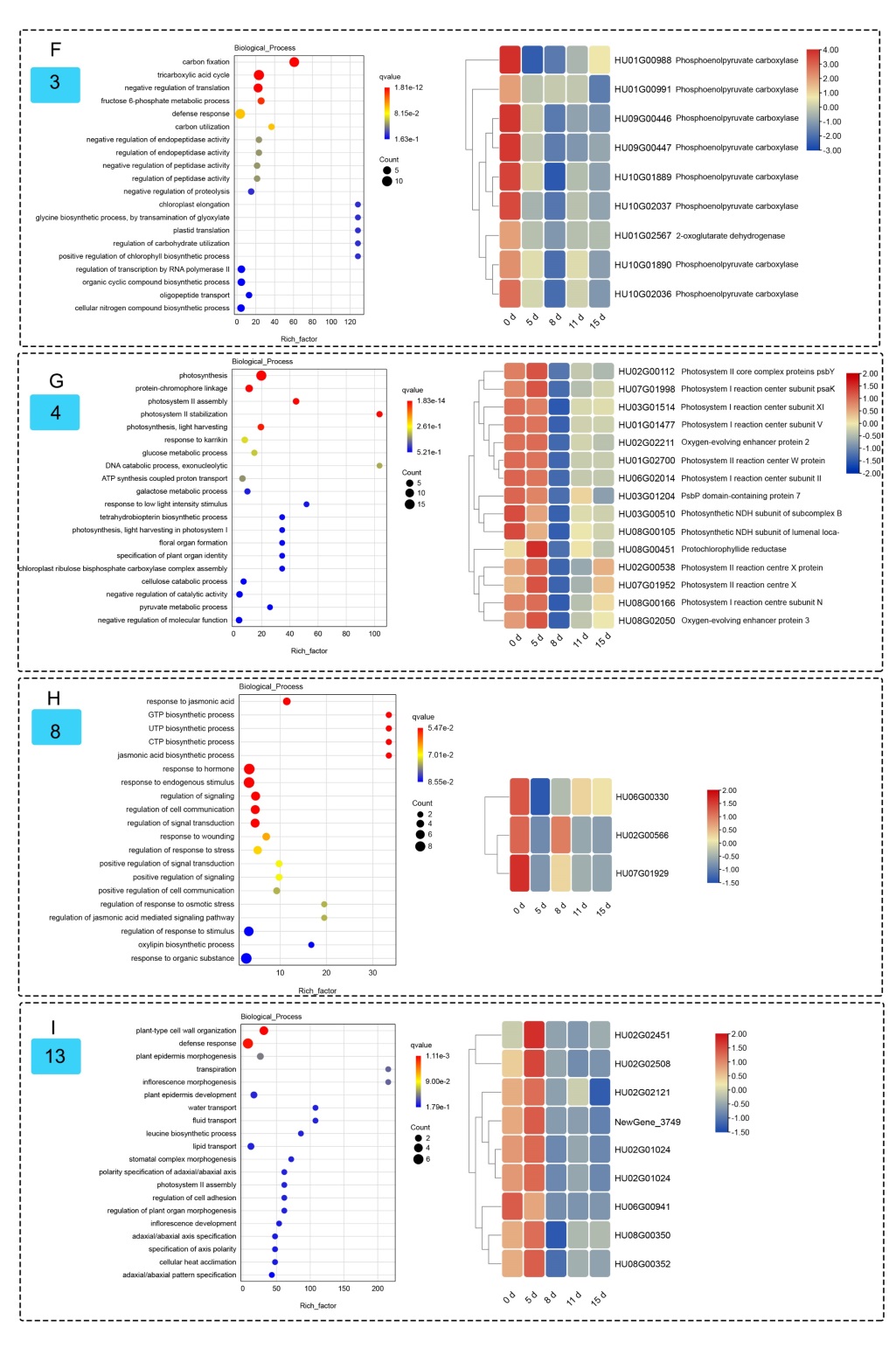

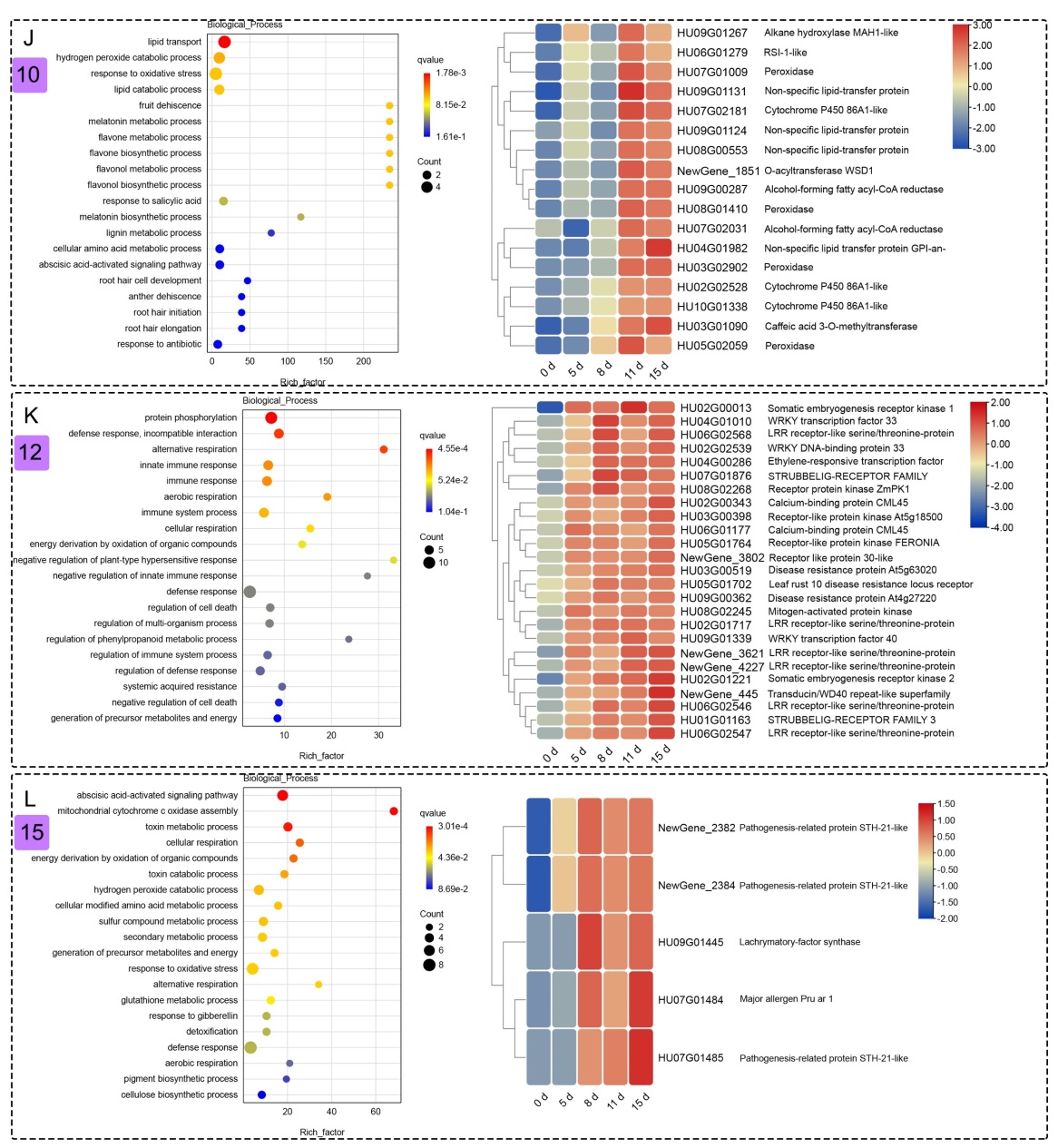


**Supplementary Figure S4.** Co-expression trend analysis. (A) Co-expression trend analysis divides the DEGs into 14 modules. Colored backgrounds indicate significant trends, white backgrounds indicate insignificant trends, and clusters of the same color indicate the same trend. (B-E) The orange color shows trends in gene expression in the early to middle stages of infection. (F-I) The blue color indicates genes with consistently decreasing expression. (J-L) The purple indicates genes with consistently increased expression.


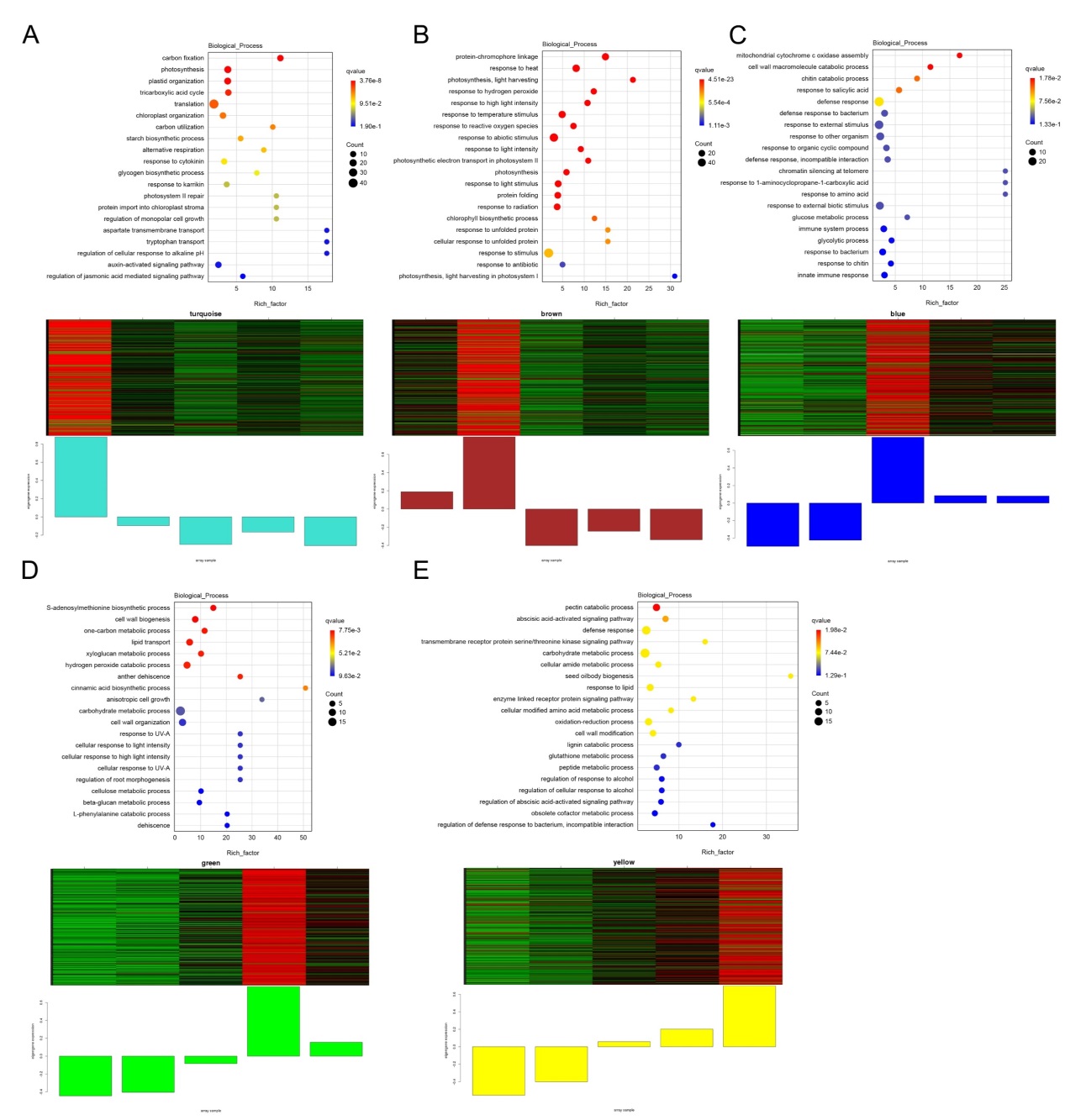
 **Supplementary Figure S5.** WGCNA divides DEGs into 5 modules, including Go enrichment analysis and heatmap analysis.
